# Fine-mapping and cell-specific enrichment at corneal resistance factor loci prioritize candidate causal regulatory variants

**DOI:** 10.1038/s42003-020-01497-w

**Published:** 2020-12-11

**Authors:** Xinyi Jiang, Nefeli Dellepiane, Erola Pairo-Castineira, Thibaud Boutin, Yatendra Kumar, Wendy A. Bickmore, Veronique Vitart

**Affiliations:** MRC Human Genetics Unit, Institute of Genetics and Molecular Medicine, University of Edinburgh, Edinburgh, EH42XU UK

**Keywords:** Genome-wide association studies, Corneal diseases

## Abstract

Corneal resistance factor (CRF) is altered during corneal diseases progression. Genome-wide-association studies (GWAS) indicated potential CRF and disease genetics overlap. Here, we characterise 135 CRF loci following GWAS in 76029 UK Biobank participants. Enrichment of extra-cellular matrix gene-sets, genetic correlation with corneal thickness (70% (SE = 5%)), reported keratoconus risk variants at 13 loci, all support relevance to corneal stroma biology. Fine-mapping identifies a subset of 55 highly likely causal variants, 91% of which are non-coding. Genomic features enrichments, using all associated variants, also indicate prominent regulatory causal role. We newly established open chromatin landscapes in two widely-used human cornea immortalised cell lines using ATAC-seq. Variants associated with CRF were significantly enriched in regulatory regions from the corneal stroma-derived cell line and enrichment increases to over 5 fold for variants prioritised by fine-mapping-including at *GAS7, SMAD3* and *COL6A1* loci. Our analysis generates many hypotheses for future functional validation of aetiological mechanisms.

## Abstract (web summary)

Here, the authors report a genome-wide association analysis for corneal resistance factor (CRF), a measure of corneal biomechanical properties relevant to a number of corneal diseases. They identify 135 loci associated with CRF and use fine-mapping and ATAC-seq in human cornea cell lines to identify putative causal variants affecting gene expression.

## Introduction

Corneal properties are clinically important^1,2^ for: disease prognosis, electing refractive eye surgery, and evaluating intraocular pressure (IOP), a critical factor in glaucoma risk assessment. Genome-wide association studies (GWAS) for measurements acquired in heathy individuals—corneal thickness^3–8^, curvature^9,10^, resistance factor and hysteresis^11^, endothelial cells shape, and density^7^—showed potential to elucidate molecular events shaping these traits and associated disease risks. Statistical methods have emerged to narrow-down the genetic variants most likely to cause observed associations, with high throughput^12^. Those can provide single-variant resolution, unlocking mechanism hypothesis, even if only for a small fraction of GWAS loci^13^. To explore this avenue, we leveraged the availability in >75-,000 UK Biobank (UKBB) participants of corneal resistance factor (CRF) measures.

CRF is an empirical measure of the corneal mechanical response to applied force. With cornea hysteresis (CH), it is increasingly computed by devices implementing bidirectional applanation tonometry to measure IOP. The corneal response to deformation is used to derive a cornea compensated IOP measure (IOPcc), which aims to remove the recognised influence of corneal thickness and viscoelasticity on Goldmann-correlated IOP measure (IOPg). CRF, by design, predicts this influence^14^, correlates more strongly with central cornea thickness (CCT) than CH^2^ and not or little with IOPcc^1,14^. CRF encompassing a CCT component—a thinner cornea should be easiest to deform—was apparent in initial CRF genetic investigations^7,11^. Three well-established CCT loci, *FOXO1*, *ZNF469*, and *COL6A1* are among the five identified genome-wide significant CRF loci^11^. CRF and CCT are concomitantly altered in Marfan syndrome patients with ectopia lentis^15^ or during keratoconus progression^1^, two conditions with corneal stroma abnomalies^16,17^. It was proposed^11^ that CRF could, like CCT^5,6^, offer genetic clues for keratoconus. Alterations during disease progression uniquely captured by CRF (after adjusting for CCT)^18,19^ make it a parameter of clinical interest in its own right. The strong CRF genetic associations at *ANAPC1* and *TCF4*^7,11^, major loci for respectively endothelial cell density^7^ and Fuchs endothelial corneal dystrophy (FECD)^20^, additionally emphasize a relevance to corneal endothelium health not prominent in the CCT GWAS results.

The cornea has three cellular layers: a stratified epithelium, a collagen-rich stroma which in human accounts for 90% of the cornea and a monolayer of cells which insures essential ion exchanges function, the endothelium^21^. The extracellular matrix (ECM) stromal components are produced by specialised mesenchymal resident cells, the keratocytes. Fittingly, many genes implicated by CCT GWAS belonged to pathways such as collagenous fibril production and regulation, or hallmarks of the mesenchymal state^6^. Stromal ECM composition being a strong determinant of cornea strength and viscoelasticity, keratocyte should also be a major target cell type for CRF-GWAS signals. Here, we identified regions of open chromatin, indicators of cis-regulatory activity, in immortalized corneal epithelial and keratocyte cell lines, using the assay for transposase accessible chromatin followed by sequencing (ATAC-seq). Overlaying these and publicly available data with the UKBB CRF-GWAS study, a homogeneous large dataset well suited for fine-mapping, identifies causal regulatory variants and regions active in fibroblastic cells as strong functional candidates. The keratocyte cell line, hTK, represents a suitable system to undertake further functional characterisation at many of those loci.

## Results

### CRF-GWAS in UK-Biobank

**Fig. 1:**
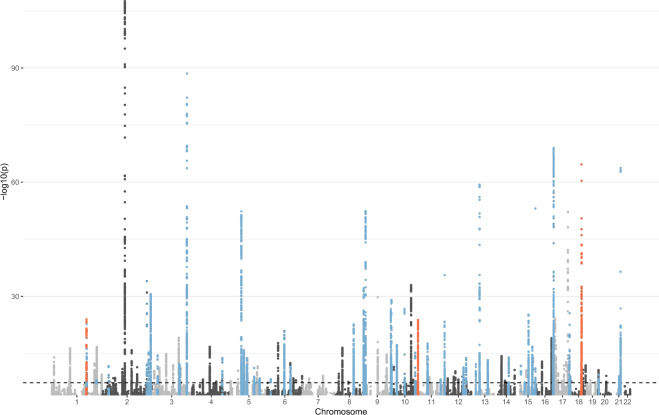
Manhattan plot of the corneal resistance factor GWAS in UK Biobank white-British participants (*n* = 76,029). The genome-wide significant threshold (*P* value = 5 × 10^−8^) is displayed by the horizontal dotted black line. Only variants with *P* value < 0.001 are represented. Genomic risk loci overlapping known central cornea thickness and Fuchs corneal dystrophy loci are indicated in blue and orange, respectively. The test statistics inflation factor λgc, 1.147, is mostly due to polygenicity (ratio (LDScore intercept − 1)/(mean(chi2) − 1) = 14.7%; LDscore intercept = 1.057(SE = 0.007)).

Genome-wide association analysis, using *N* = 76,029 UKBB participants of white-British ancestry, yielded 135 loci harbouring variants significantly associated (*P* value < 5 × 10^−8^) with corneal resistance factor (Fig. 1, Supplementary Data 1). Those include 251 potentially independent variants based heuristically on pairwise linkage disequilibrium measure r^2^ less than 0.1 (Supplementary Data 1), suggesting multiple association signals at several loci. Variant effects were consistent between this analysis and a GWAS performed in a smaller set of European non-British participants (*N* = 10,130) (Supplementary Fig. 1, Supplementary Data 2), with the exception of strong but opposite-direction effects for the lead, low-frequency variant, rs112108520 at the *ETS1* locus. Forty eight out of the 135 loci map CCT loci, identified in independent and smaller studies^6–8^, including thirteen which have been associated with keratoconus risk^5,6,8,22^ (Supplementary Data 1). Using the International Genetics Glaucoma Consortium (IGGC)^6^ CCT summary statistics for participants of European ancestry and linkage disequilibrium (LD) score regression method^23^, the genetic correlation between CRF and CCT is 70% (SE = 5%). Colocalisation tests further support shared causal variants underlying these two traits at all but two of the 27 loci significantly associated with CCT in the IGGC European subset (Supplementary Data 3). Signals at *FNDC3B* (loci 31) and *RXRA*-*COL5A1* (locus 68) were attributed to both CRF and CCT, but declared not to be the same. Both loci are complex with several independently associated SNPs^6^, which may contribute differently to both traits. Two CRF novel loci, *ATP1B1-LINC00970* (locus 5) and *SLC25A22* (locus 78), together with the previously reported *TCF4* (locus 126), map to FECD risk loci^24^, with close match of association plots (Supplementary Fig. 2). The FECD risk alleles of representative SNPs at these three sites were all associated with decreasing corneal resistance, and their effects on FECD risk appear correlated with their effect on CRF (Supplementary Fig. 2).

More than half of the CRF loci (*N* = 72) have been reported as IOPg loci in analysis of a larger, fully overlapping UKBB sample^25^. In the white-British sample, the phenotypic correlation between CRF and IOPg was 59.2% and the genotypic correlation 73.44% (SE = 1.72%). In agreement with expectation, the phenotypic correlation with IOPcc was lower, 5.3%, and fewer CRF loci (*N* = 18) overlap with reported loci^11^. Interestingly, two of those, *ATP1B1- LINC00970* and *ANAPC1* have not been reported for IOPg, but for FECD^24^ and endothelial cell density, respectively^7^. This suggests an influence of corneal features on IOPcc, qualitatively different to that affecting IOPg.

Candidate CRF-GWAS target genes selected by the FUMA algorithm^26^ are listed Supplementary Data 4 alongside the selection criteria (physical location, chromatin interaction evidences, or/and eQTL data). Using this gene list, the top two biological pathways implicated by geneset enrichment test are GO_cc, go_proteinaceous_extracellular_matrix (enrichment *P* value 1.92 × 10^−16^) and go_extracellular_matrix (*P* val 4.17 × 10^−15^). Both enrichments were highly significant (multi-test corrected 5% threshold of 0.05/10673 = 4.68 × 10^−6^) and in line with expectation of a major role for the stroma in corneal resistance.

### Refinement of CRF association signals using statistical methods

To avoid haplotype structure due to close relatedness, these analyses were performed on the subset of unrelated white-British participants, yielding a smaller but still substantial number of genome-wide significant loci, *N* = 115, including all those noted as overlapping FECD and keratoconus risk loci in the larger analysis (Supplementary Data 5). The subanalysis newly flags seven loci (association *P* value nearing genome-wide significance threshold), none of which previously known to affect cornea structure. Conditional and joint multiple-SNP (CoJo) analysis implemented in the program GCTA^27^ defined multiple causal signals at 22 loci yielding 149 independent associated lead variants (Supplementary Data 6). The stepwise selection of predictor variants implemented being suboptimal in regions with multiple signals in linkage disequilibrium, we also use the exhaustive search for joint combination of alleles method implemented in FINEMAP^28^. This suggested 186 independent causal signals, largely concordant with those defined by CoJo (Supplementary Data 7). Since the Bayesian method implemented in FINEMAP returns strength of evidence for a variant to contribute to each signal, these causal signals could be fine-mapped to sets of variants, with 95% probability. Sets were named after the variant with the highest posterior inclusion probability (PIP) (Supplementary Data 7); their size ranged from 1 (at loci 16, *AC078954.2*, and 92, *IGF1*) to 293 (at locus 40, *CWC27*). The full list of variants included in these 95% credible sets is given Supplementary Data 8, with PIP alongside the causal evidence support given as Bayes factor (BF).

### Subset of highly likely causal variants

Fifty-five variants (Supplementary Data 9) with high PIP (>60%) and/or very strong evidence for being causal (log10(BF) > 3) were examined in greater detail. They comprised five coding variants (Supplementary Data 10), all missense with a high (>20) CADD score, a measure of the predicted deleterious consequence of the amino acid substitution^29^. All have been previously prioritized in relation to CCT^7,8^ or keratoconus^11^, apart from rs77583146, p.Gly165Arg in *WNT10A*. This latter is 146 bp away from another low-frequency missense variant, rs121908120, reported for CCT and keratoconus risk^22^, identified here as an independent functional variant candidate. All proteins implicated, WNT10A, ABCA6, GLT8D2, FBN2, and ADAMTS17, have reported function relevant to corneal development or maintenance (Supplementary Data 10).

**Fig. 2:**
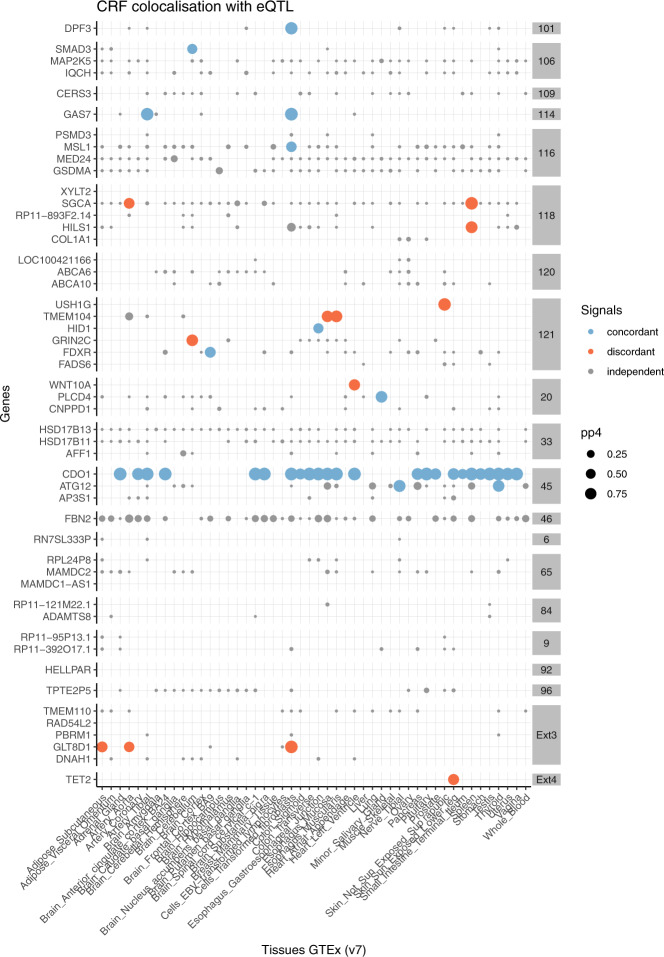
Colocalisation with GTEx v7 eQTL signals at prioritized corneal resistance factor loci. Genes: candidate target genes based on GTEx eQTL look-up for prioritized candidate causal variants at CRF loci (grey box). Each dot denotes significant association signals for both CRF and GTEx v7 GWAS, colour-coded as independent (grey) or identical (blue if CRF increasing allele increases gene expression, red if it decreases it). The dot size is proportional to the probability of a colocalisation (pp4); pp4 > 0.75 denotes strong evidence in favour of colocalisation.

The majority of prioritised candidate causal variants (50/55), representing 46 independent credible sets at 34 loci, were noncoding (Supplementary Data 9). Over half (in 28 credible sets across 26 loci) are significantly associated with gene transcription or alternative splicing levels in at least one tissue/cell type from public depositories (Supplementary Data 9). To explore whether some of those variants could be causally associated with corneal resistance by modulating gene expression, we applied colocalisation tests for CRF and cis-eQTL using available GTEx^30^ v7 full summary statistics. Colocalisation is often detected across several tissues for a given gene (Fig. 2), lending support that if observed it could also occur in the cornea—a tissue unrepresented in GTEx. The biological sample with the most evidence of colocalisations (*N* = 5) was “Cells-transformed fibroblasts”. Whilst more than one gene was implicated at several loci, a unique candidate target gene was supported at six of the investigated loci: *DPF3* (at locus 101), *SMAD3 (*at locus 106*), GAS7* (at locus 114), *MSL1* (at locus 116), *GLT8D1* (at locus Ext3), and *TET2* (at locus Ext4). The pertinence of the function for all six genes in the corneal context is summarised Table 1.

**Table 1:** Six noncoding highly likely causal variants for CRF associations colocalising with a single gene expression signal.

Prioritised and eQTL	Locus	MAF	PIP	log10(BF)	COLOC pp	Implicated gene	Protein function
rs759509948	101	0.360	0.495	3.324	0.916	*DPF3*	Transcription regulator, histone acetyl-lysine reader with zinc and tandem PHD fingers. Component of tissue and stage specific chromatin remodelling complexes with roles in neuro (nBAF^68^), and muscle developments^69^. Pitx2, a major TF in cornea development, is a Dpb3b target gene in a mouse myoblast cell line^70^. DPF3 is expressed in primary cultured cornea fibroblasts (PCFs) and downregulated in PCFs from granular cornea dystrophy patients with mutations in TGFBI^71^.
rs12913547	106	0.209	0.415	3.117	0.539	*SMAD3*	Transcription factor involved in TGFβ signal transduction. Several *SMAD3* mutations cause the autosomal dominant connective tissue disorder Loeys–Dietz Syndrome 3 (OMIM: 613795), ocular features of which include reduced central cornea thickness^72^
rs62257617^a^	Ext3	0.052	0.297	3.320	0.959	*GLT8D1*	Shares high homology and UDP-glucose:glycoprotein glucosyltransferase potential with GLT8D2^73^, implicated by a fine-mapped prioritised coding variant at separate locus (Supplementary Data 10).
rs9913911	114	0.375	0.842	3.532	0.998	*GAS7*	Growth-arrest specific 7 gene expression was first identified in serum-starved cultured murine fibroblasts^74^. Gas7 plays an important role in neural crest and neural-cells-derived tissues development in zebrafish^75^.
rs141144358	116	0.344	0.978	4.411	0.608	*MSL1*	Component of a complex responsible for histone H4 acetylation at lysine 16, which by promoting open chromatin structure impacts on gene transcriptional regulation^76^.
rs10010325^b^	Ext4	0.486	0.489	3.195	0.68	*TET2*	Methyl cytosine dioxygenase 2 which drives DNA demethylation to cytosine hydroxymethylation, e.g. at enhancers during cellular reprogramming^77^ including hypoxia-induced^78^ʼ one.

Gene expression data used for colocalisation tests correspond to all cells/tissues represented in GTExv7.

*MAF* minor allele frequency, *COLOC pp* posterior probability of signals to be identical, *PIP* posterior probability of inclusion to list of candidate variants causing association signal, *log10(BF)* log10 of the Bayes factor quantifying how likely the variant is to be causal rather than non causal.

^a^PIP other variant in same credible set with high log10(BF), rs186144945, not significant eQTL.

^b^Other variant in same credible set with high log10(BF), rs2903385, is also an eQTL.

For the subset of 30 noncoding SNPs singled-out by a PIP > 0.6, we examined the potential for transcription factor (TF) binding site disruption (summarised in Supplementary Data 11 and presented in full Supplementary Data 12). Two TFs whose binding strengths were predicted to be significantly altered at the most sites (7 out of the 30 investigated sites) were REST and RXRA (Table 2). Chance expectations for such results were estimated to be 9.7% and 7.34%, for REST and RXRA, respectively (Supplementary Note 5). Notably, amongst the seven loci with a prioritised causal variant predicted to affect REST binding affinity, *FNDC3*, *RXRA-COL5A1*, and *FOXO1* are keratoconus loci and *ADAMTS8* and *COL6A1*, were recently suggested to be^11^.

**Table 2:** Prioritised CRF causal candidate variants with potential to disrupt a REST or RXRA binding site.

TF	variant	Ref/Alt	Altered motif	Motif source^a^	Stronger bind (*P* value)	Stronger bind CRF association	MM	Annot	Locus
REST	rs3132302	A/G	ggcggagtgagcAg	ENCODE REST_disc8	G (3.4 × 10^−4^)	Decreasing	0.9315	intergenic *RXRA*-*COL5A1*	68
	rs58933977	T/G	Tgtgcacacaatgg	ENCODE REST_disc8	G (1.7 × 10^−4^)	Decreasing	0.9472	intergenic *MEG8-DIO3OS*	102
	rs7635832	T/G	ctcagcacatttTtt	ENCODE REST_disc1	G (4.8 × 10^−4^)	Decreasing	0.7364	intronic *FNDC3*	31
	rs79126727	G/A	Ggccctgt	ENCODE REST_disc6	A (6.1 × 10^−5^)	Decreasing	0.9297	intronic *FTCD*	135
	rs182804464	C/G	cttctCctcagcttgctgt	ENCODE REST_known3	G (4.3 × 10^−4^)	Decreasing	0.6829	intronic *COL6A1*	135
	rs74948688^a^	C/T	ccatCacacccagc	ENCODE REST_disc9	C (2.3 × 10^−4^)^a^	Increasing	0.9108	intronic and 3’*FOXO1*	96
	rs2875238	T/C	gccactggttcTggt	ENCODE REST_disc1	T (2 × 10^−4^)	Increasing	0.7685	intronic and 5’ *ADAMTS8*	84
RXRA	rs2035835	G/C	cGccatct	ENCODE RXRA_disc5	G (3 × 10^−5^)	Decreasing	0.9832	intronic *OTOP2*	121
	rs34869^a^	G/C	aagggtcggaggaGatg	ENCODE RXRA_known6	C (1 × 10^−4^)^a^	Increasing	0.7247	5’ *CDO1*	45
	rs10868299	C/A	gggtaCcttagagacca	ENCODE RXRA_known8	A (1.2 × 10^−4^)	Increasing	0.8288	intergenic *C9orf135*-*MAMDC2*	65
	rs74948688^a^	C/T	tttccatCac	ENCODE RXRA_disc4	T (4.1 × 10^−4^)^a^	Decreasing	0.9043	intronic and 3’*FOXO1*	96
	rs143038218	T/A	cgTgagtaaa	ENCODE RXRA_disc3	T (3 × 10^−4^)	Increasing	0.9395	intergenic *UBIAD1*-*PTCHD2*	1
	rs6081765	A/G	caAgttca	ENCODE RXRA_known4	A (1.7 × 10^−4^)	Increasing	0.98899	Intergenic *PDYN-STK35*	130
	rs7863424^a^	G/A	agagccagagGgga	SwissRegulon	G (2.3 × 10^−4^)^a^	Decreasing	0.7977	intronic *GLIS3*	63

In silico prediction of significant differential binding for reference (Ref) and alternate (Alt) allele at fine-mapped prioritised SNPs was computed using transcription factor motif weight matrices from various sources (Motif Source) using MotifBreakR.

*MM* motif match as percentage of perfect match.

^a^Denotes a corresponding transcription factor occupancy peak in the ENCODE 3 ChIP-seq data survey of 338 factors in 130 cell types.

RXRA is encoded by a gene flanking one of the CRF-GWAS locus (*RXRA*-*COL5A1*). Three out of the seven highly likely causal variants predicted to disrupt RXRA binding are located within a RXRA ChIP peak in one of the tissues surveyed by the ENCODE project, including the variant intronic to *FOXO1*, which is also predicted to alter REST binding and also within an ENCODE REST ChIP peak (Table 2). In addition to RXRA, ten TFs for which binding sites could be disrupted in at least two highly likely causal polymorphic sites are encoded by candidate GWAS target genes (Supplementary Data 4 and 11). For one of those, TCF4, the prioritised variant predicted to alter its binding, rs192498625, locates in the vicinity of the TF encoding gene (intronic one *TCF4* isoform, upstream other isoforms). Supporting a (self) regulatory role, among all the tissues examined by the Roadmap Epigenomics Consortium^31^, the histone modification H3K27ac, considered as a hallmark of active enhancers, is detected around this site, exquisitely in (H1 derived) mesenchymal stem cells^32^.

Of the prioritised noncoding eQTL variants linked to a unique target gene (Table 1), only rs9913911, predicted to modulate expression of *GAS7*, was examined (SNP and PIP > 0.6) for transcription factor binding disruption potential. Several factors could be affected (Supplementary Data 12), including the aryl hydrogen receptor class with perfect match between the underlying sequence (with reference A allele at rs9913911) and binding motifs.

### Cornea cells-derived ATAC-seq datasets for enrichment analysis

Enrichment of CRF-GWAS variants in regulatory regions of the genome was apparent using annotations generated in a broad range of tissues by the ENCODE, GENCODE, or Roadmap Epigenomics projects^33^. Enrichment was the lowest for regulatory features derived from blood cells and fetal brain tissues and high (>2.5 fold), for those from lung, heart, fetal muscle, eye, skin, fetal thymus, fibroblasts (Supplementary Fig. 3). That from fetal muscle, which contains fibroblasts and myoblasts of mesenchymal stem cells origin, was the strongest, around 3.3 fold with association *P* value cut-off of 10^−6^. These enrichments support GWAS variants impacting prominently regulatory activity in connective tissues and fibroblasts in particular.

**Fig. 3:**
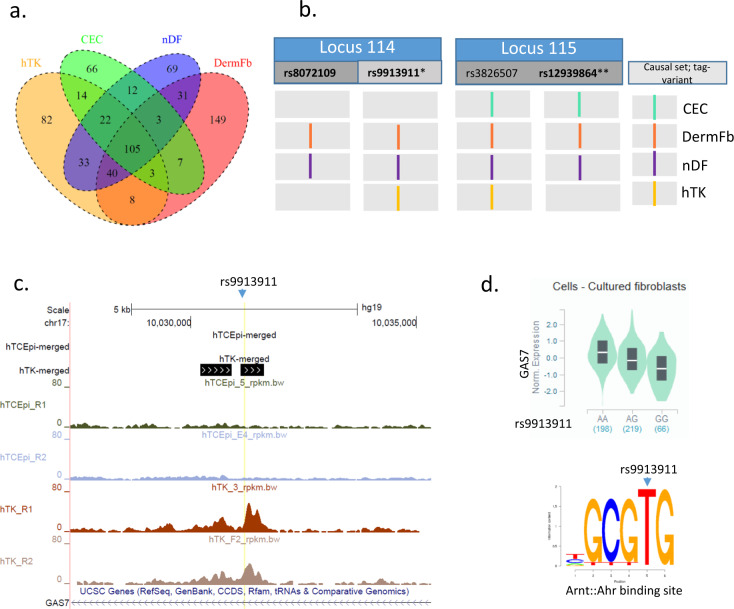
Regulatory genomic annotations for associated CRF-GWAS variants. **a** Overlap of open chromatin regions mapped to GWAS variants (*P*-value threshold > 10^−8^ or tagging, r^2^ > 0.8, variants) across four ATAC-seq datasets significantly enriched in CRF-GWAS variants. Cell origins: adult cornea epithelium primary tissue (CEC); skin fibroblasts (DermFb); neonate skin fibroblasts (nDF); immortalized corneal keratocytes hTK. **b** Overlap details at two CRF loci. Variants named are those selected for enrichment analysis based on *P*-value threshold (tag variant), bold indicates that they belong to 95% credible sets of causal variants and fall in OCR themselves (*) or tag another credible set variant that do. **different credible set variants map to OCR: rs4646785 in CEC and nDF, rs12939864 in DermFb. **c** ATAC-seq profiles in immortalised corneal epithelial and stromal cell lines (respectively, hTCEpi and hTK, each in duplicate) around variant rs9913911, prioritised causal variant at locus 114, credible set 2. Screenshot from UCSC genome browser with annotation coordinates used in enrichment analysis in top tracks. **d** eQTL data from GTEx v8 and predicted significantly disrupted transcription factor binding motif at rs9913911 (T > C).

We then investigated the levels of open chromatin regions enrichment, using published and purposely generated ATAC-seq datasets, in order to examine that for keratocytes, the specialised fibroblasts that populated the corneal stroma. Available datasets selected (Supplementary Data 13) include those derived from: primary cornea epithelial cells (CEC), cranial neural crest cells (cornea endothelial cells and keratocytes having a neural crest origin), adult and neonate skin fibroblasts (DermFb and nDF, respectively), primary retinal pigmented epithelium (RPE), immortalised retinal pigmented epithelium cells (RPE_CellLine) and two, a priori negative, controls, lymphoblastoid and myelogenous leukemia cells. We performed ATAC-seq in the telomerase-immortalised human cornea cell lines hTK and hTCEpi, derived respectively from stromal^34^ and epithelial^35^ corneal tissues. The greatest enrichment for the CRF-GWAS results, using association *P* value threshold of 10^−8^, was 1.82 and significant (*p* = 4.28 × 10^−8^) in open chromatin regions (OCR) of the keratocyte cell line, hTK, after those present in the hTCEpi cell line have been subtracted out, here named hTK-specific OCR (Table 3). Of note, the enrichment is sensitive to the ATAC-seq peak calling and subtraction method adopted (Supplementary Note 3). When considering hTK regulatory features without filtering out those present in hTCEpi, enrichment is decreased to a level similar to that of adult or neonate dermal fibroblasts, all of which were significant. Enrichments using open chromatin regions derived from the two blood derived cells lines were not significantly different than those expected by chance, nor were those derived from the epithelial cells hTCEpi or RPE. The enrichment in primary cornea epithelium cells (CEC)-derived features, although lower than those from the fibroblastic cells, was significant and hence cautions on hTCEpi representing faithfully the primary tissue state. The open chromatin regions annotated by CRF-associated variants overlap across the four significantly enriched datasets (Fig. 3a), with detailed information in Supplementary Data 14. Whether those variants belong to 95% credible causal sets narrows down candidate causal regions as illustrated Fig. 3b for two loci. At locus 115, *ALDH3A1*, two distinct OCR are mapped but only one, in epithelial corneal cells and dermal fibroblasts, by a credible set variant. At locus 114, *GAS7*, a variant from each of the two independent credible causal sets map distinct regulatory regions, the former active in dermal cells only, the latter in all fibroblastic cells (nDF, DermFb, and hTK). ATAC-seq profiles from the in-house datasets around this second region show it to be inaccessible in hTCEpi cells (Fig. 3c). The variant tagging this region, rs9913911, associates with *GAS7* transcription levels in skin-derived cultured fibroblasts (Fig. 2 and Fig. 3d) with the CRF-and gene expression-increasing allele, T, displaying strongest arnt::ahr binding potential (Fig. 3d).

**Table 3:** Cell-specific open chromatin regions enrichment analyses for CRF-associated variants.

		Enrichment							
ATAC-seq annotation	Tag variants	OR	CI95_L	CI95_U	*P* value	NAnnotThr	NAnnot	NThr	N
Cranial neural crest cells CNCC_donor2	GWAS *P*-value > 10^−8^	1.45	1.14	1.83	2.10E-03	77	53,001	3692	4,428,625
	Likely causal BF > = 3	0.98	0.13	7.61	9.82E-01	1	53,812	26	4,429,172
Adult skin fibroblasts DermF	GWAS *P*-value > 10^−8^	1.57	1.37	1.80	**5.98E-11**	251	172,059	3692	4,428,625
	Likely causal BF > = 3	3.36	1.29	8.79	1.35E-02	7	173,842	26	4,429,172
Neonate dermal fibroblasts nDF	GWAS *P*-value > 10^−8^	1.54	1.34	1.78	**2.04E-09**	236	160,782	3692	4,428,625
	Likely causal BF > = 3	2.24	0.78	6.47	1.35E-01	5	162,447	26	4,429,172
Primary cornea epithelium CEC	GWAS *P*-value > 10^−8^	1.42	1.21	1.66	**1.03E-05**	194	143,339	3692	4,428,625
	Likely causal BF > = 3	1.91	0.61	6.01	2.67E-01	4	145,008	26	4,429,172
Cornea epithelium cell line hTCEpi	GWAS *P*-value > 10^−8^	1.07	0.90	1.27	4.22E-01	155	144,979	3692	4,428,625
	Likely causal BF > = 3	0.71	0.16	3.22	6.59E-01	2	146,425	26	4,429,172
hTCEpi-specific^a^	GWAS *P*-value > 10^−8^	0.67	0.47	0.95	2.65E-02	34	49,520	3692	4,428,625
	Likely causal BF > = 3	0.997	0.13	7.76	9.97E-01	1	50,324	26	4,429,172
Cornea keratocyte cell line hTK	GWAS *P*-value > 10^−8^	1.51	1.31	1.73	**1.23E-08**	243	168,314	3692	4,428,625
	Likely causal BF > = 3	2.85	1.05	7.73	3.92E-02	6	170,143	26	4,429,172
hTK-specific^a^	GWAS *P*-value > 10^−8^	1.82	1.47	2.25	**4.28E-08**	107	60,099	3692	4,428,625
	Likely causal BF > = 3	5.68	1.81	17.80	2.88E-03	4	61,152	26	4,429,172
Myelogenous leukemia cell line K562	GWAS *P*-value > 10^−8^	1.39	1.09	1.76	7.17E-03	74	52,657	3692	4,428,625
	Likely causal BF > = 3	0.90	0.11	7.05	9.20E-01	1	53,554	26	4,429,172
Lymphoblastoid cell line LCL_NA18504	GWAS *P*-value > 10^−8^	1.25	1.01	1.54	3.93E-02	99	77,529	3692	4,428,625
	Likely causal BF > = 3	1.45	0.32	6.57	6.28E-01	2	78,638	26	4,429,172
Retinal pigmented epithelium RPE_donor2^b^	GWAS *P*-value > 10^−8^	1.40	1.13	1.73	1.90E-03	95	67,560	3692	4,428,625
	Likely causal BF > = 3	2.55	0.70	9.23	1.55E-01	3	68,519	26	4,429,172

Enrichment and significance was assessed using GARFIELD^33^ with associated variants (tag variants) thresholded based on P value or Bayes factor for causality. Significant enrichments are highlighted in bold.

*BF* log10 of the Bayes factor, *NAnnotThr* the number of variants passing CRF association threshold criteria, which are annotated to an ATAC-seq feature, *NAnnot* the total number of variants annotated to feature, *NThr* the total number of independent variants passing CRF association threshold criteria, *N* the total number of variants analysed following LD pruning.

^a^Results vary depending on peak-calling algorithm used as presented in Supplementary Note 3.

^b^Three independent datasets were tested—RPE_Cell line yield nearly identical results, RPE_Donor3 had lower Nannot and less significant *P* value.

Enrichment is higher, but did not reach significance threshold, when only using the much reduced set of fine-mapped variants with strong causality support (Table 3). The highest enrichment was obtained for hTK-specific open chromatin region (OR = 5.68, 95% CI [1.81–17.8], p = 2.9 × 10^−3^), closely followed by those for DermFb and hTK. In total, eight highly likely causal variants tag OCRs in either of these tissue/cells (Supplementary Data 15). Five locate within an OCR themselves, including the three variants proposed to cause association via modulation of *SMAD3*, *GAS7*, and *MSL1* expression (Table 1). The prioritized intronic *SMAD3* and intergenic between *COL6A1* and *COL6A2* variants, locate within regions of accessible chromatin in the corneal fibroblast cell line (Supplementary Fig. 4). For all but one of the selected tag variants outside a regulatory annotation, at least one linkage disequilibrium-tagged variant within OCRs belong to the credible set, with PIP ranging from 0.014 to 0.15 and log10(BF) from 1.56 to 2.36.

## Discussion

GWAS analysis in the UKBB white-British sample led to 130 novel genome-wide significant CRF loci, many with multiple independent causal signals, providing a rich resource to investigate regulatory mechanisms. Although not strictly replicated given the current absence of a sample of similar size, the signals show concordance of effects in European non-British UKBB participants. We focused particularly on identifying and describing the subset of genetic variations with a strong statistical support of being causal. This set is reduced given pervasive linkage disequilibrium in the genome and leaves unexamined many associations that may be more prominent and important for an understanding of diseases or key regulatory pathways. However, this subset provides a foundation for mechanistic insights. We showed that some of those could be pursued in the TERT-immortalised keratocyte cell line hTK, which we selected as a human cell line a priori the most relevant to corneal thickness and resistance.

The multidimensionality of the CRF measure complicates interpretation of results. While CRF was designed to capture cornea properties impacting on IOPg measures, it may still be influenced by true IOP. Reciprocally, our data support that IOPcc is not devoid of relationship with corneal features, in particular those other than CCT, which agrees with observations that IOPcc, not IOPg, is sensitive to change in FECD^36^. Glaucoma GWAS results should in principle help disentangling effects on IOP from those on cornea per se. However, properties of the corneal cells, keratocytes in particular, are shared by other ocular tissues important in glaucoma pathology: the trabecular meshwork cells or the connective tissue of the optic nerve head^37,38^. Strong associations with IOPg, IOPcc and glaucoma for the *GAS7* variant for example suggest that its effect on CRF is via IOP. Yet, its annotation to open chromatin region in corneal fibroblastic cells show that biological horizontal pleiotropy, a common feature of genetic variant effects, cannot be disproved. Disentangling CRF and IOPcc relationship using Mendelian Randomisation (MR) is one focus of CRF and CH UKBB GWAS^39^ analyses published whilst our manuscript was under review. Pitfalls and assumptions underlying MR techniques^40^ make advancing our understanding of genetic effects a pressing need.

CRF causal variants appear well suited to inform on corneal stromal cell biology as evidenced by enrichment analyses with the ECM pathways topping the list of biological genesets and with regulatory annotations generated in fibroblasts and mesenchymal tissues topping that of regulatory annotations. Connective tissue disorders genes *FBN2*, *ADAMTS17*, and *SMAD3* affected by fine-mapped variants, as well as lower enrichment of regions active in cornea epithelial cells compared to that in fibroblastic cells lines, and improved enrichment when OCR shared with hTCEpi are removed from the hTK dataset, also support this notion.

The CRF-associated variants enrichment of OCR annotations in hTK was, arguably, not substantially greater than that obtained using skin fibroblast cell lines, and both display the most significant enrichments. This may not be too surprising as we expect many of the GWAS variants to be concerned with maintenance or establishment of the fibroblastic state, and Mendelian disorders associated with thin cornea (e.g. Brittle Cornea and Ehlers–Danlos Syndromes) are also often characterised by general connective tissue dysfunction^41–43^. Yet, effects of genetic variants could be tissue-specific and broadening cell-type repertoire is warranted for mechanistic insights. Prioritised causal variants falling into OCRs in both the dermal and corneal fibroblastic cell lines, rs8127032 (locus 135), intergenic between *COL6A1* and *COL6A2*, or the intronic variants at *SMAD3* (locus 106) are appealing candidates to start exploring mechanism and cell-specificity questions further. Type VI collagen is a major component of the human cornea stroma^44^ with a suggested role in cornea tensile strength^45^. It is a component of the ECM in muscle, vessels and skin—tissues which display the most prominent features of type VI collagenopathies. Recent exome sequence analysis in UKBB^46^ reported significant large effects on CRF of a burden of rare loss of function coding variants in *COL6A1;* rs8127032 could shed light on cis-regulatory control of this gene. Drawing on the ever-increasing regulatory annotations of the genome, particularly on experimentally defined trans-acting factors binding sites, could further shape precise hypotheses to be tested. Two of the three prioritized variants at *SMAD3* locate within AP-1 components (FOS/JUN/JUND/FOSL2) binding sites in the ENCODE 3 data. AP-1 and SMAD3 are important mediators of TGF-β dependent gene regulation, including that of genes involved in ECM homeostasis^47,48^.

Given that the activity of many regulatory elements will be development stage and/or environment dependent—situations unlikely to be all encapsulated in available cells and tissues, we also attempted to complement enrichment analyses of regulatory features using in silico prediction of the disruptiveness of prioritised variants on TF binding sites. Suggestion of RXRA as potential link between several of the prioritised noncoding variants is supported by importance of retinoic acid signalling during cornea development and isolated keratocyte phenotypes including ECM composition^49^. Functional follow up will be required to ascertain relevant links especially as we used a lenient threshold for motif matching. This was chosen for discovery purpose given the lack of accuracy of in silico predictions compounded with possible functional range of transcription factors binding-affinities.

Our analyses converged nicely within locus 114 to pinpoint one noncoding causal candidate, rs9913911, giving clues as to how it could exert its effect—modulating transcription levels of *GAS7* by affecting the binding of a trans-acting factor. The basic helix-loop-helix transcription factors Ahr, ARNT (HIF1β), or ARNT2, which mediate response to developmental and environmental stimuli, appeared strong contenders to be affected trans-acting factors. Down-regulation by hypoxia of *GAS7*, *ARNT2*, and *HIF1α* transcript levels in monocytes^50^ opens a possible hypoxia response connection.

Overall, our analysis provides many leads to plan hypothesis-driven experimental analyses, which are ultimately required to demonstrate causality and function of CRF-GWAS variants. It also provides ground for further investigations as in addition to the limitations already alluded to, including a focus on transcription factor-dependent causal mechanisms, we did not investigate corneal endothelial cells, an important target tissue for corneal dystrophies for which CRF appeared to be a good endophenotype, and the cornea immortalized-cell lines studied were derived from a unique donor (*N* = 1).

## Methods

### Study population

The UK Biobank is a large-scale prospective study established by the Medical Research Council, Department of Health, Wellcome Trust, Scottish Government and North-West Regional Development Agency^51^. The study was conducted with the approval of the North-West Research Ethics Committee (Reference: 06/MRE08/65).

Between 2006 and 2010, close to 500,000 people (273,467 female/229,175 male) were recruited. Genome-wide genotype data and imputations on the full set were made available to the international community with primary genotype quality controls and analyses such as ancestry grouping and detection of close kinship (http://www.ukbiobank.ac.uk/wp-content/uploads/2014/04/imputation_documentation_May2015.pdf and ref. ^52^). A detailed description of the study and its access process are available online (http://www.ukbiobank.ac.uk/resources/).

Phenotypes and genotypes used in this manuscript were obtained through approved application number 19655. Analyses presented are those performed with the largest ethnic subset of participants, that of European ancestry, passing phenotypic and genotypic quality controls. The main analysis was carried out in participants of the white-British ancestry subset defined by Bycroft et al.^52^.

A nonoverlapping subset of UKBB participants of European ancestry, among those who self-reported “other white background” or “Irish” ancestry, was used to perform independent genetic analysis. A genetically homogeneous subset for those participants was created following the method used to define the subset of white-British ancestry. Briefly, participants genetically European, based on the principal component analysis (PCA) using 1000 Genomes project Phase I reference samples performed by Bycroft et al.^52^, were selected if within five standard deviations from the self-reported European sample mean for each of the three first principal components (discriminating European, Asian and African ancestry super-groups). Multidimensional scaling was then performed based on the kinship matrix derived from the subset of individuals with corneal measures and not selected as white-British, with LD pruned genotyped markers of call rate >99%, MAF > 1%, and not in regions of high LD^52^ using KING^53^. Eight participants with outlying values on PC 5 and 6 were removed, leaving 10130 individuals. Final clustering was performed using the 500 reference samples of European origins from the 1000 Genomes Project Phase3 which were not closely related (of 3rd or greater degree of kinship) and with markers intersecting those present in the selected sample. Final PCs (*N* = 10) to use as covariates for the association analysis were generated by projecting the selected samples (*N* = 10,130) to the PC space of reference samples using KING^53^.

### Ocular response analyser measures

Eye examinations were performed on 127,453 individuals, close to 25% of the UK Biobank participants, at six assessment centres, and include bidirectional applanation tonometry using a Reichert Ocular Response Analyser (ORA). The ORA produces parameters derived from the measured applanation pressures, p1 and p2 where *p*_1_ is the pressure at which the cornea flattens as air-pressure is applied, and *p*_2_ the pressure at which applanation reoccurs after the air-pressure is released^14^. A Golmann-correlated IOP measure (IOPg) is calculated as average of p1 and p2, and a corneal hysteresis measure (CH), is calculated as the difference between the two pressures. Corneal resistance factor (CRF) and a cornea compensated IOP measure, IOPcc, are derived as linear combinations of p1 and p2 following proprietary formulae. Those are devised to minimize the changes in IOP measurements before and after LASIK refractive surgery^14^. All measures are expressed in mm of mercury.

CRF analysis was performed on the average of the left and right eye measurements, following some filtering of the available measures. The 1041 UK Biobank participants with extreme inter-ocular differences (greater than population mean difference + 3 standard deviations) were removed. Additionally, 5001 participants were excluded as having self-reported or being linked to an ocular condition that could affect the measurements accuracy. Those were self-reported recent eye surgery (code 5181 in corresponding UK Biobank data-field), refractive laser surgery (code 5325), cataract surgery (code 5324), glaucoma high pressure surgery or laser treatment (codes 5326 and 5327), corneal graft surgery (code 5328), eye injury (code 5419); and the following electronic health records (data-fields 41202-41205):keratoconus (ICD10: H18.6; ICD9: 3716) or cornea disorders (ICD10: H18.4-9).

Finally, the samples failing the centrally performed quality controls for heterozygosity or/and missingness^52^, or having a mismatch between self-reported and genotype-derived gender or showing putative sex chromosome aneuploidy as well as individuals who have withdrawn from the study at the time of analysis were removed.

A total of 76,029 white-British participants and 10,130 European not white-British were thus available for GWAS analyses. The CRF distribution and characteristics in the white-British dataset are presented in Supplementary Note 1, including effect of age and sex which were adjusted for in the GWAS analysis.

### GWAS analysis

Single-variant associations were performed by testing for an additive allelic effect at each (HRC + UK10K) imputed genotypes in the white-British subset defined by Bycroft et al.^52^, and, separately, in the independent sample of European not white-British UKBB participants described in the Study population section. Only the well imputed (INFO > 0.6) and common to low-frequency (MAF > 0.5%) variants were tested. The primary GWAS with all measured individuals were performed using a linear mixed model, accounting for population structure and (cryptic) relatedness, implemented in the software BOLT_LMM v1.3^54,55^. Covariates fitted in the model were: age, sex, assessment centre, genotyping array, genotyping batch, and principal components of ancestry (20 for the white-British analysis, 10 for the European not white-British analysis). Fine-mapping analyses were performed on GWAS results obtained from the restricted set of measured individuals who were not closely related (pairwise kinship coefficient greater than 0.025 calculated using KING^53^), amounting to *N* = 72,301 individuals. GWAS there was performed using PLINK2^56^.

### Preliminary functional annotation and gene mapping

SNPs annotations, gene mapping, and geneset enrichment test were performed with the functional mapping and annotation of genome-wide association studies (FUMA) platform v1.3.5^26^. UKBB release 2, with a subset of 10,000 white-British participants, was selected as the linkage disequilibrium (LD) reference for the annotations. Those included the determination of independent lead SNPs in a locus, defined heuristically by FUMA using a LD measure r^2^ cut-off of 0.1. Loci were defined by the coordinates of variants in LD (r^2^ > =0.6) with lead variant (lowest *P*-value) or independent lead variants and merging of LD blocks located less than 250 kb to each other. The SNP2GENE function in FUMA was used to annotate putative target genes in an exhaustive manner. A first annotation include physical mapping. Other candidate genes, which can locate outside loci limits, are those whose expression has been significantly associated with GWAS variants based on a comprehensive source of eQTL repositories at time of query (August 2019-GTEx, eQTLGen, Common Mind Consortium, Blood eQTL, BIOS, and BRAINEAC) or based on chromatin interaction maps available at time of query (PsychENCODE, FANTOM5, Hi-C data GSE87112 and Giusti-Rodriguez_et_al_2019) with tissue or cell type where interactions have been performed. The references for those resources compiled by FUMA can be found at https://fuma.ctglab.nl/links.

The geneset enrichment test implemented in MAGMA^57^ v1.06 was also performed within FUMA, with genesets from MsigDB v6.2. Significance threshold is based on Bonferroni correction.

### GWAS signals colocalisation

Bayesian colocalisation analysis as proposed by Giambartolomei et al.^58^ was performed using the default priors of the *coloc.compute* function of the R package *gtx* 2.1.6 (https://github.com/tobyjohnson/gtx). This framework provides posterior probabilities for each possible scenario within the region tested: neither trait has a genetic association (pp0), only trait1 has (pp1), only trait2 has (pp2), both trait1 and trait2 are associated but with different causal variants (pp3), and both traits share causal association signal(s) (pp4). A pp4 > 75% indicates strong evidence that both GWAS signals colocalise.

### Multiple signals detection and fine-mapping

The genome-wide complex trait analysis (GCTA) software package (C++), version 1.9.1 beta3, implements a multi-SNP stepwise model selection, which can indicate multiple functional variants within a region and estimates SNP effect conditional on effect of nearby selected variants^27^. The stepwise model selection (--cojo-slct) was run with the default *P*-value threshold of 5 × 10^−8^, collinearity threshold of 0.9, with genotypes (hard calls from imputed dosages as implemented in plink2) and phenotype files used for input. A computationally efficient Bayesian alternative variant selection method implemented (C++) in FINEMAP^28^, v1.3.1, was also performed. The shotgun stochastic search algorithm was used (-sss argument) using the number of causal SNPs with associated posterior probabilities determined from a prerun with the maximal number of causal SNPs set to 10 (--n-causal-snps = 10). The data required for each investigated locus are the corresponding GWAS summary statistics and a regional LD matrix. This latter was computed from the imputed genotypes posterior probabilities stored in UKBB bgen files using LDstore^59^. FINEMAP reported a set of CRF-associated variants for each detected independent association signal, with 95% probability of containing the causal variant—so called 95% credible set. Attached to each variant are two useful metrics to rank the candidate variants: its posterior probability to be included in the credible set (PIP), with note that two variants in complete LD will have the same PIP score, and a Bayes factor (log10 scale) quantifying how likely the variant is to be causal rather than non--causal, with log10 Bayes factor (log10(BF)) greater than 2 deemed decisive evidence.

### TF binding sites prediction

We used motifbreakR^60^ to identify variants predicted to strongly alter binding of a transcription factor (TF) based on position probability matrices (PPM). Two sources of TF motifs encompassing 14 public collections (including JASPAR, HOCOMOCO, ENCODE, HOMER and FactorBook) were used (MotifDb and motifbreakR_motif). Analysis was carried out with the program default settings apart from the *P*-value threshold to declare TF binding site matching either of the allelic configuration set to 5 × 10^−4^ and the relative entropy scoring method set to information content algorithm (method = “ic”) as performed in ref. ^61^.

Accurate *P*-values for each allele match were calculated for a subset of variants of interest using the function calculatePvalue() implementing the algorithm developed by Touzet and Varre^62^. All but three prioritised variants, those corresponding to indels rs141144358, rs200584273, and rs5877786, could be analysed. To derive probability of the findings by chance, sampling of GWAS variants matched to the query SNPs based on allele frequency, number of SNPs in LD, distance to nearest gene and gene density^63^ was generated 10,000 times as described in more details in Supplementary Note 5.

### ATAC-seq

Assay for transposase accessible chromatin followed by sequencing (ATAC-seq) was performed on two cornea immortalised cell lines generated by Prof J Jester and collaborators and kindly gifted to us by Dr Che Connon (University of Newcastle): the hTCEpi cell line derived from primary human cornea epithelial cells^35^ and the hTK cell line derived from primary cornea keratocytes^34^, resident cells of the cornea stroma. hTCEpi and hTK cells were cultured respectively in KGM^TM^-2 basal medium with KGM^TM^-2 Single-Quots^TM^ supplements (Lonza) and Dulbecco’s Modified Eagle Medium (DMEM) supplemented with 10% fetal bovine serum, with addition of 1% penicillin and streptavidin in both media. Two replicates of hTCEpi and hTK cell batches were harvested at 70–90% confluence, and 50,000 cells processed per experiment. Nuclei preparation and transposase digestion followed the ATAC-seq protocol described in ref. ^64^ and are further detailed in Supplementary Note 2, together with library preparation and sequencing. We obtained over 75 million usable uniquely mapped paired-end reads for each sample (min 78,877,410– max 192,169,752) following removal of reads mapping to the mitochondrial genome, or to more than one genomic region or duplicated (Supplementary Note 2). The genomic coverage of replicates (two independent cultures) was highly reproducible with Pearson’s correlation coefficients of 0.958 and 0.987 respectively for hTCEpi and hTK cells. Peak calling was performed using the findPeaks command from the HOMER software package^65^ as detailed in Supplementary Note 2, and replicates combined following algorithms detailed Supplementary Note 3. Sets of ATAC-seq peaks present in corneal keratocyte but not in the epithelium derived cells lines and vice versa were also derived using a few calling variations (Supplementary Note 3).

Published datasets from relevant or control human cells were obtained from NCBI Gene Expression Omnibus (GEO) and their quality checked in a similar way to that used for the in-house datasets. Briefly, the original fastq reads were downloaded and sequences aligned using bowtie2 (v 2.3.4.1-1), as paired ended alignments for all but the K562 cell lines data (Supplementary Data 13). In general, read numbers were small for individual datasets and pooling was performed to create a better set for confident peaks calling (Supplementary Data 13). Fragments length plots, on which the Supplementary Data 13 fragment quality criterion is based, are presented Supplementary Note 4. Resemblance between tissues/cells ATAC-seq sets was evaluated by unsupervised clustering of the original BAM files using the plotCorrelation option of the deeptools suite^66^ in Python and illustrated Supplementary Fig. 5.

### Tissue-/cell-specific functional annotation enrichment analysis

We use the GWAS Analysis of Regulatory or Functional Information Enrichment with LD correction (GARFIELD)^33^ to perform annotation enrichment analysis. It tests using logistic regression whether associated variants (or tagged variants) map significantly more frequently to regions of interest than the nonassociated variants. The method takes into account variations in LD patterns and gene density across the genome as well as the correlations between functional annotations when multiple sets of annotation are tested together. Genomic locations tagged by the GWAS SNPs at different *P*-value thresholds were investigated, as well as those tagged by the restricted set of identified SNPs with strong statistical support for being causal (log10(BF) greater or equal to 3). To perform feature enrichments for those SNPs, a file was created with dummy P-values, above threshold (>10^-8^) for all variants except those of interest which were allocated an under threshold P-value.

Analysis was first carried out using the functional annotations provided within the package, those from Roadmap Epigenomics^31^ and Encyclopedia of DNA Elements (ENCODE)^67^. We then performed enrichment analysis for cell-specific chromatin accessible regions by using custom annotations corresponding to the ATAC-seq datasets described in the ATAC-seq section.

We prepared GARFIELD variants-related input files anew so that all the variants analysed (Imputation Score > 0.6 and MAF > 0.005) and their pairwise LD measures matched the GWAS dataset (UK Biobank white-British ancestry). Those input files contain information on the distance to the nearest transcription start site for each variant, the lists of SNPs that can be clumped together (r^2^ > 0.1) for the first step of greedy pruning of GWAS SNPs and the lists of SNPs that are proxies to the GWAS SNPs (r^2^ > 0.8) for scoring overlap with an annotated feature. Enrichment *P*-values reported by GARFIELD are adjusted with respect to multiple testing using a Bonferonni correction and an effective number of tests carried out based on the correlation structure of the annotation features tested. For the ATAC-seq datasets tested, which were analysed all together, the significance threshold (false positive rate of 5%) was 0.00055.

*URLs***:** UK Biobank Access Management System, http://www.ukbiobank.ac.uk/register-apply/; ENCODE, https://www.encodeproject.org/; FUMA, https://fuma.ctglab.nl/; GCTA, http://cnsgenomics.com/software/gcta/#Overview; GENCODE https://www.gencodegenes.org/; MotifBreakR, https://www.bioconductor.org/packages/release/bioc/html/motifbreakR.html; UCSC genome browser, https://genome.ucsc.edu/; GARFIELD, https://www.ebi.ac.uk/birney-srv/GARFIELD/; GEO, https://www.ncbi.nlm.nih.gov; GTEx portal, https://gtexportal.org/home/; Roadmap Epigenomics Consortium http://www.roadmapepigenomics.org/; KING http://people.virginia.edu/~wc9c/KING/kingpopulation.html; MSigDB https://www.gsea-msigdb.org/gsea/msigdb/index.jsp.

## Supplementary information

Supplementary Material

Description of Additional Supplementary Items

Supplementary Data

## Data Availability

The UK Biobank resource, open to all bona fide health researchers, is available upon request through their access management system. The summary statistics for the analysis of CRF in participants of white-British ancestry can be downloaded from https://doi.org/10.7488/ds/2944. Novel ATAC-seq data generated in the hTCEpi and hTK cells have been deposited on GEO under accession GSE150064.
