# Supplementary material for: Fine-mapping and cell-specific enrichment at corneal resistance factor loci prioritize candidate causal regulatory variants
Source: Commun Biol. 2020 Dec 11;3:762. doi: 10.1038/s42003-020-01497-w (PMC7732848; doi:10.1038/s42003-020-01497-w)
Supplement: Supplementary file 1 — Supplementary Material [file 42003_2020_1497_MOESM1_ESM.pdf]

Supplementary Information files

Fine-mapping and cell-specific enrichment at corneal resistance factor loci prioritize candidate causal regulatory variants

Xinyi Jiang[1,#], Nefeli Dellepiane[1,#], Erola Pairo-Castineira[1,#], Thibaud Boutin[1,#], Yatendra Kumar[1#], Wendy A. Bickmore[1] and Veronique Vitart[1*]

[1] MRC Human Genetics Unit, Institute of Genetics and Molecular Medicine, University of Edinburgh, Edinburgh, EH42XU, UK

[#]These authors contributed equally

Supplementary Notes pp 2-10

Supplementary Figures pp 11-15

Supplementary Note 1. Cornea resistance factor distribution and summary statistics for the white-British UK Biobank participants (N=76029) analysed

Cross-population CRF measures show close to normal distribution.

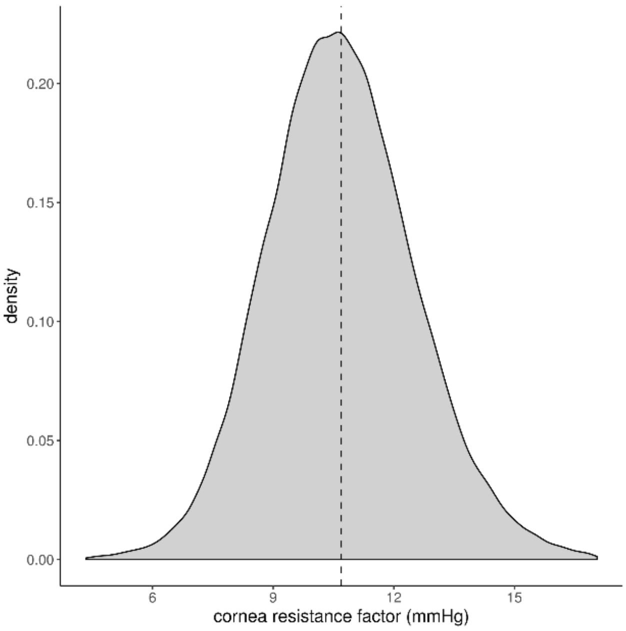

Summary statistics for the CRF measures are tabulated below

	Count	Mean (mm Hg)	SD	Range
Overall	76029	10.69	1.83	4.34 – 17.05
Male	35267	10.53	1.84	4.34 -17.05
Female	40762	10.82	1.82	4.40 – 17.05

Effects of age (per decade) and sex (male) on CRF are highly significant and displayed below

Covariate	Mean effect in mm Hg (standard error)	P-value
Age (decade)	-0.144 (0.008)	$<2 \times 10^{-16}$
Sex (Male)	-0.293 (0.013)	$<2 \times 10^{-16}$

Supplementary Note 2. Assay for Transposase-accessible chromatin followed by sequencing (ATAC-seq).

2.1 Nuclei preparation and transposase digestion.

50000 cells were collected and washed with 100uL cold PBS by spinning at 500rcf for 5min at 4°C. Cells were then lysed by resuspension in 100uL ice-cold ATAC-seq lysis buffer (10mM Tris-Cl ph7.4, 10mM NaCl, 3mM $MgCl_2$, 0.1% NP40), followed by incubation on ice for 30 minutes with occasional mixing with the pipette. Nuclear pellet was isolated by spinning at 1000rcf for 5 minutes at 4°C and resuspended in 50uL transposition mix (2.5uL Tn5 transposase in 10mM Tris ph8, 5mM $MgCl_2$, 10% Dimethylformamide). Tagmentation reaction was incubated at 37C for 35min in a thermomixer at 700rpm. Tagmentation was stopped by immediate purification using MinElute Reaction Clean up Kit (Qiagen Cat No:28204) leading to elution of tagmented DNA 11uL of water.

2.2 Library preparation and quality assessment

Libraries were prepared as described previously[1] with a universal forward primer and a reverse primer with a different index for each experiment. A 32ul PCR reaction was prepared containing: the tagmented DNA (10ul), NEBNext High-Fidelity 2X PCR Master Mix (M0541S, NEB), 50X SYBR green for PCR (163795-75-3, Sigma-Aldrich) and 10uM each of forward and reverse nextera adaptors. PCR was initiated by incubating at 72C for 5 min in LightCycler-480 real time PCR machine (Roche). Amplification was achieved by 10-12 cycles of 98°C for 10 seconds, 63°C for 30 seconds and 72°C for 1 minute. PCR reactions were stopped before reaching a plateau, recovered and supplemented with water to a final volume of 50ul. We purified the PCR reactions using 50ul (ratio 1:1) of the Ampure-XP beads (cat# 10136224, Beckman Coulter) to remove excess adaptors using manufacturer's protocol and eluted DNA in 20uL water. DNA concentration was checked with dsDNA High-sensitivity assay (cat# Q32854, Qbit) followed by estimation of digestion profiles using double-stranded DNA high sensitivity assay on Bioanalyzer (Agilent) to make sure that Tn5 produced a nucleosomal ladder pattern.

2.3 Next Generation Sequencing and data processing

Libraries were sequenced on Illumina Hi-Seq platform in paired-end mode for 75 cycles. Sequencing reads were assessed for quality using FastQCv0.11.4[2] and adaptor sequences were trimmed using cutadapt (V2.0) in paired-end mode. Reads were aligned to UCSC hg19 assembly using bowtie2 (default parameters with *-X 2000 --very-sensitive --no-discordant --no-mixed --no-unal*). PCR duplicates were removed using PICARD and reads mapping to mitochondrial genome and ENCODE blacklisted regions[3] were removed using samtools[4]. The number of total usable reads obtained were ~79 and 90 million reads for the hTK cell line replicates R1 and R2 and ~114 and 192 for the hTCEpi cell line replicates R1 and R2 respectively. Finally, we converted alignments to BED format and extracted fragment lengths using bedtools suite[5]. The fragment length distribution of the ATAC-seq reads is shown below:

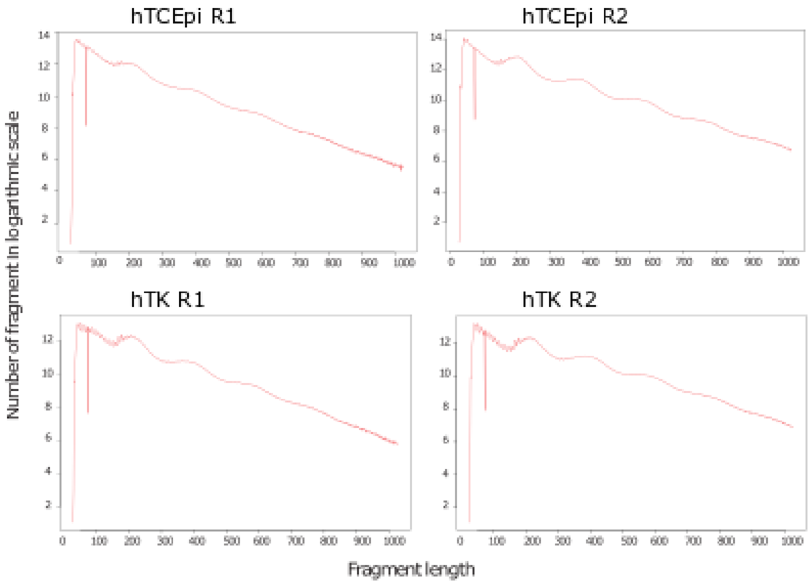

2.4 Visualization and identification of accessible regions

BED files were used to create tag directories in HOMER suite[6] followed by generation of bedgraph files using HOMER's *makeUCSCfile* and subsequent conversion into bigwig format with *bedGraphToBigWig* program[7]. Bigwig files were visualized in UCSC genome browser[8]. We called peaks using HOMER's findPeaks program with parameters suitable to locally focussed peak calls against the background computed from a large local window (*-localSize 50000 -size 150 -region-minDist 300 –fragLength*

0 –tbp 0). We calculated log2 scaled normalized read counts in 5kb continuous windows across the genome and calculated the Pearson’s coefficient for replicates (0.958 and 0.987 respectively for hTCEpi and hTK replicates) which indicated very high reproducibility among replicates.

Supplementary Note 3. ATAC-seq Peak calling options and impact on enrichment analysis

3.1 Combining peak calls for the replicates.

ATAC-seq signals were combined using the intersect command from bedtools[5] software. Two rules were applied. In rule 1 (merge), intersect option –u was used- peaks found in one replicate only were excluded from the combined peaks file, whereas overlapping peaks from the two replicates were combined in one “combined peak”, followed by the merge command. In rule 2 (intercept) intersect option –u –f 0.5 –r was used- overlapping peaks between the two replicates required a reciprocal overlap of at least 50% to be included in the final combined peaks file.

3.2 Calling cell type-specific open chromatin peaks

Annotations for open chromatin regions (OCRs) that were present in hTK and not in hTCEpi and vice versa were performed using the commands intersect and substract from bedtools in a new set of subtract rules 1 and 2 respectively. Four different “specific data set” were created for each of hTK and hTCEpi: **A-** combined replicates using rule 1 followed by substract rule 1- when there is a 50% or more overlap between peaks found in the two cell lines, the peaks are not included as “specific peaks” (option: -f 0.5), when the overlap between cell lines is less than 50%, the peak of hTCEpi or vice versa cell line is not subtracted and the peak found in hTK or vice versa cell line is directed to the output file “cell line specific peaks”, **B-** combined replicates using rule 2 followed by substract rule 1, **C-** combined replicates using rule 1 followed by substract rule 2- excludes peaks that show any overlap between the two cell lines (option: -v) from the output cell line specific file, **D-** combined replicates using rule 2 followed by substract rule 2.

3.3 Influence of peak calling algorithm on OCR GARFIELD enrichment analysis

Enrichment analysis was run using GARFIELD[9] with an extended set of data including all the options described in 3.1 and 3.2 for combining replicates and calling the hTK and hTCEpi specific ATACseq peaks. The datasets derived from the same raw data are highly correlated therefore their inclusion should have little impact on the enrichment P-value significance threshold calculated internally. Enrichment analysis results for the hTK and hTCEpi OCRs shown below illustrate the impact of the peak calling algorithm.

Variation	Annotation	OR	CI95_lowe	CI95_uppe	Pvalue	NAnnotTh	NAnnot	NThresh	N
Combining replicates	hTK-rule1	1.51	1.31	1.73	1.23E-08	243	168314	3692	4428625
	hTK-rule2	1.49	1.28	1.73	2.43E-07	208	142765	3692	4428625
Specific peak calling	hTK Specific-A	1.72	1.43	2.06	4.9E-09	147	89010	3692	4428625
	hTK Specific-B	1.43	1.17	1.75	0.000408	121	84495	3692	4428625
	hTK Specific-C	1.82	1.47	2.25	4.28E-08	107	60099	3692	4428625
	hTK Specific-D	1.36	1.07	1.73	0.012604	85	60477	3692	4428625
Combining replicates	hTCEpi-rule1	1.07	0.90	1.27	0.421891	155	144979	3692	4428625
	hTCEpi-rule2	1.19	1.00	1.42	0.051487	144	120712	3692	4428625
Specific peak calling	hTCEpi Specific-A	0.75	0.55	1.03	0.074827	43	56362	3692	4428625
	hTCEpi Specific-B	0.81	0.56	1.17	0.256582	30	36522	3692	4428625
	hTCEpi Specific-C	0.67	0.47	0.95	0.026539	34	49520	3692	4428625
	hTCEpi Specific-D	0.88	0.64	1.21	0.421551	41	46065	3692	4428625

NAnnotThresh -the number of independent variants using the CRF association threshold $p=10^{-8}$ which are annotated with the given feature, NAnnot – the total number of annotated variants, NThresh - the total number of independent variants selected using association threshold and N- the total number of variants analysed following LD pruning.

Supplementary Note 4. ATAC fragments length distribution of evaluated publicly available datasets. More details and GEO ids are given Supplementary Data 11. An ATACseq experiment was deemed successful if the fragments length distribution plot displays decreasing and periodical peaks corresponding to the nucleosome-free regions (< 100 bp) and mono-, di-, and tri-nucleosomes (~ 200, 400, 600 bp, respectively)

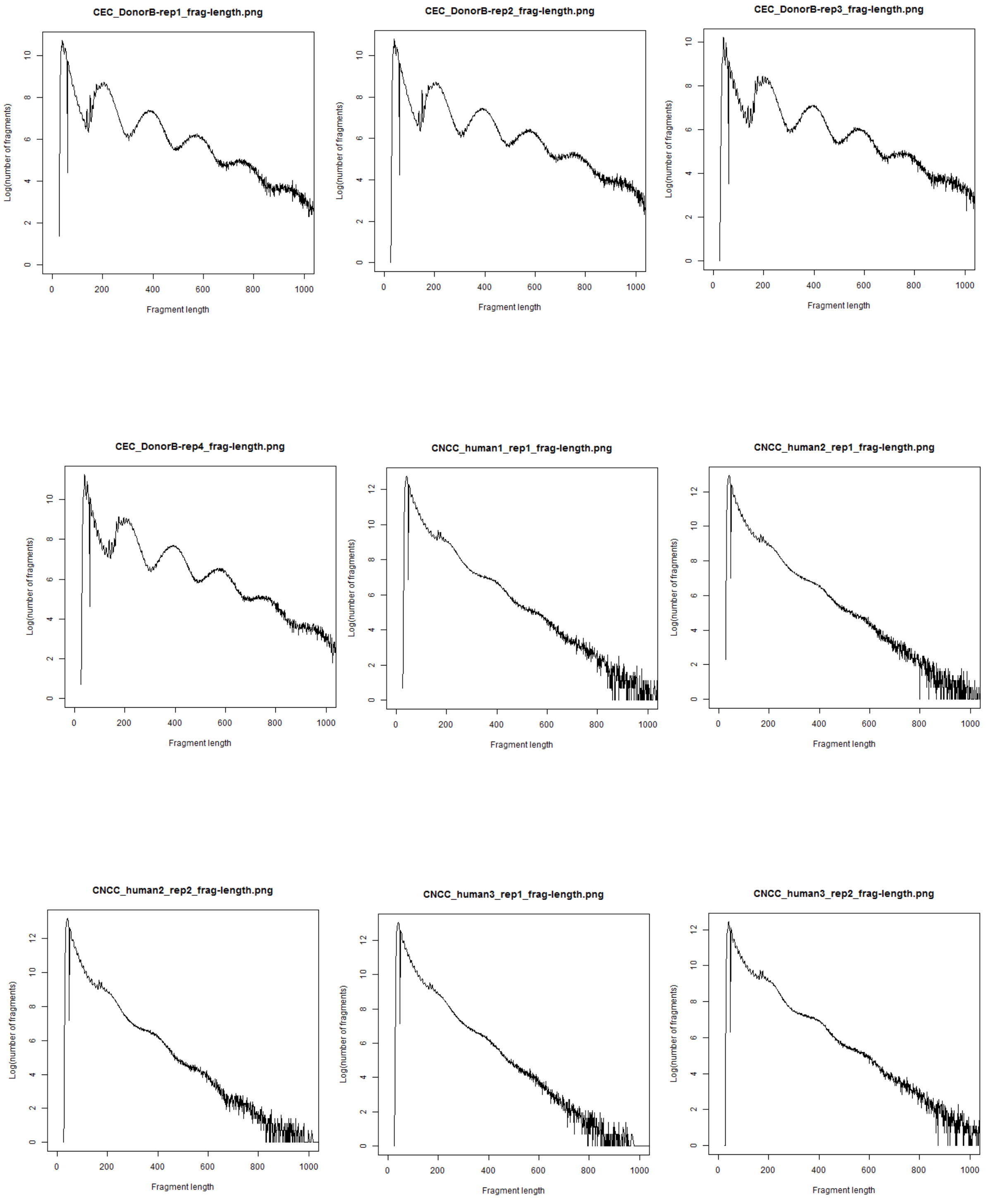

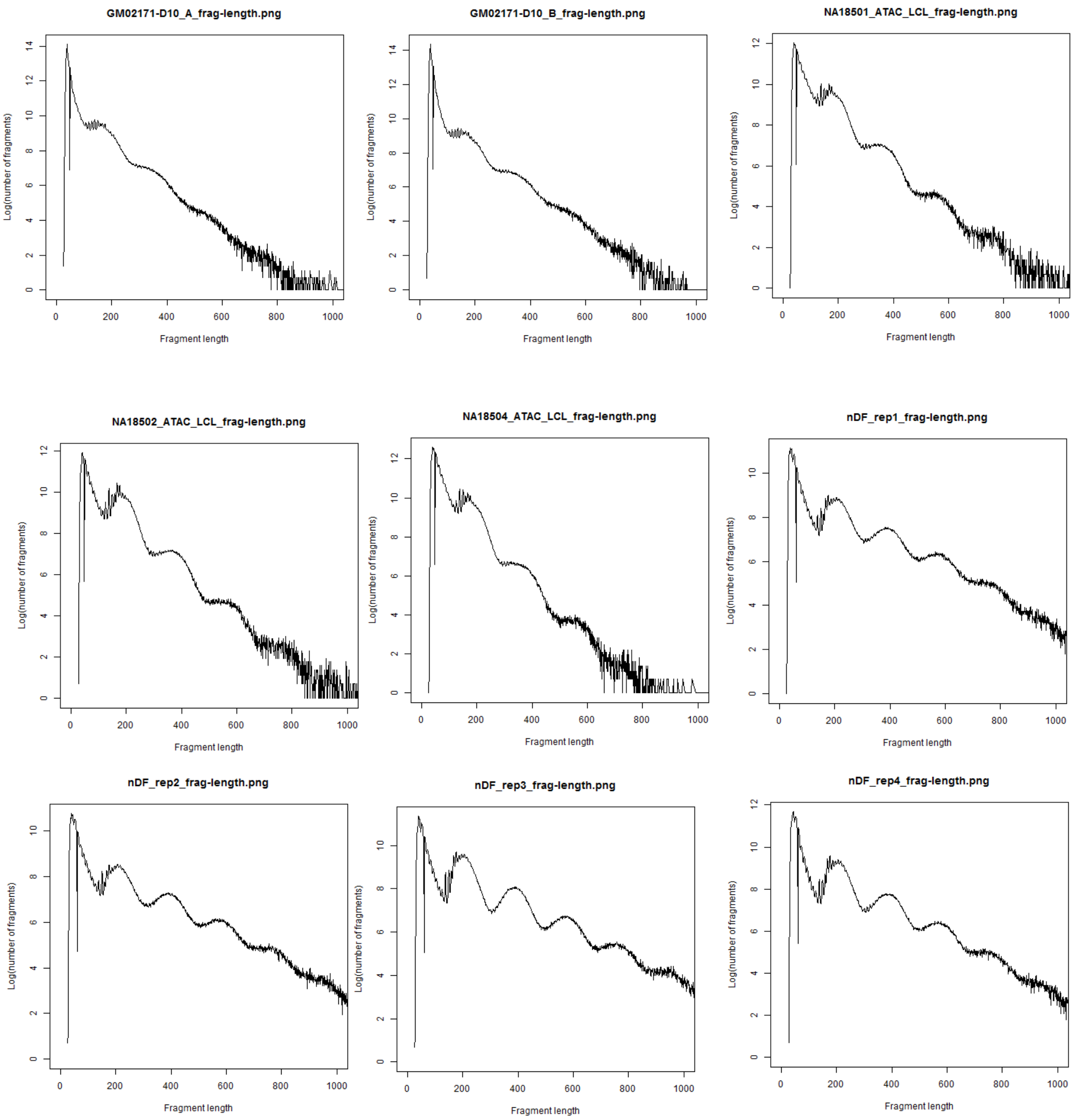
GM02171-D10_A_frag-length.png
GM02171-D10_B_frag-length.png
NA18501_ATAC_LCL_frag-length.png
NA18502_ATAC_LCL_frag-length.png
NA18504_ATAC_LCL_frag-length.png
nDF_rep1_frag-length.png
nDF_rep2_frag-length.png
nDF_rep3_frag-length.png
nDF_rep4_frag-length.png
Log(number of fragments)
Fragment length

Supplementary Note 5. Null distribution for REST and RXRA predicted binding sites.

10 0000 sets of SNPs of similar genomic properties as the tested 30 SNPs were obtained using SNPsnap (https://data.broadinstitute.org/mpg/snpsnap/) using default settings. SNPs that were not in the SNPsnap repository (N=8) or for which insufficient number of matches could be found (N=1, rs34869) were replaced by neighbouring variants with similar regulome DB score (https://www.regulomedb.org/regulome-search/), or/and matching MAF or/and in LD with tested SNP. Not all sets returned had 30 SNPs which could be analysed by motifbreakR, hence analysis for the null distribution was done on all sets, extrapolating linearly results obtained to that of a set of 30 SNPs as well as on the 242 complete sets as sensitivity analysis.

Number of matching SNP per set	19	20	21	22	23	24	25	26	27	28	29	30
Set count	2	7	29	95	275	644	1279	2003	2373	2013	1038	242

For each set, motifBreakR was run using both MotifDb and MotifbreakR_motif databases and number of independent SNPs predicted to strongly alter REST and RXRA bindings scored.

Distribution of scores using the 242 sets with 30 matching SNPs is shown below.

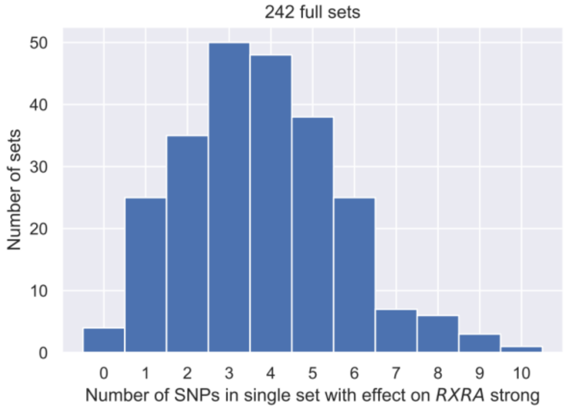

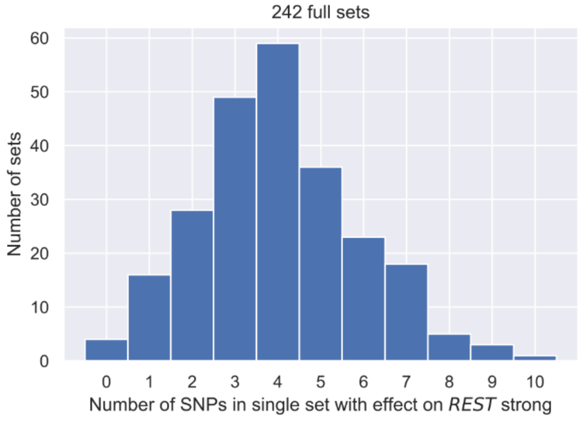

Distribution of scores using all 10000 sets, extrapolating results to set size of 30 SNPs is shown below.

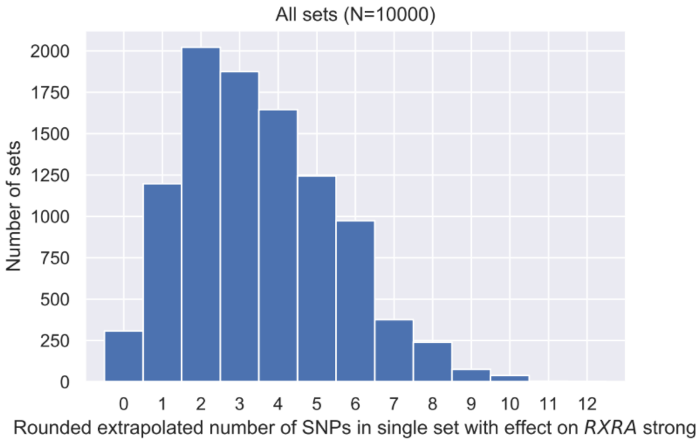

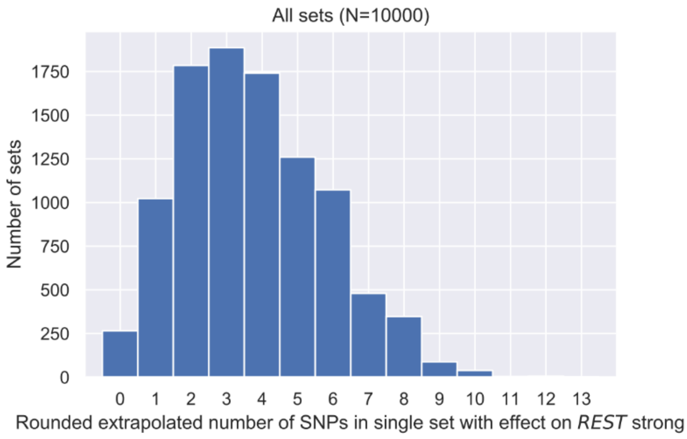

Supplementary Figure 1. Correlation of effect sizes for CRF between UKBB white-British (N= 76029) and UKBB European non-white British (N=10130) for the lead associated variants in the largest study. A. For the 135 index variants with the lowest P-values (P-value < 5 10^{-8}) within CRF-associated loci. B. For all 251 variants genome-wide significant and potentially independent (based on low linkage disequilibrium with index SNP) listed Supplementary Data 1. Black line represents the linear best fit, with equation reported within graph, the dotted line represents y=x. Effect estimates on the x and y axis are given in mmHg. Complete summary statistics corresponding to these graphs are listed Supplementary Data 2.

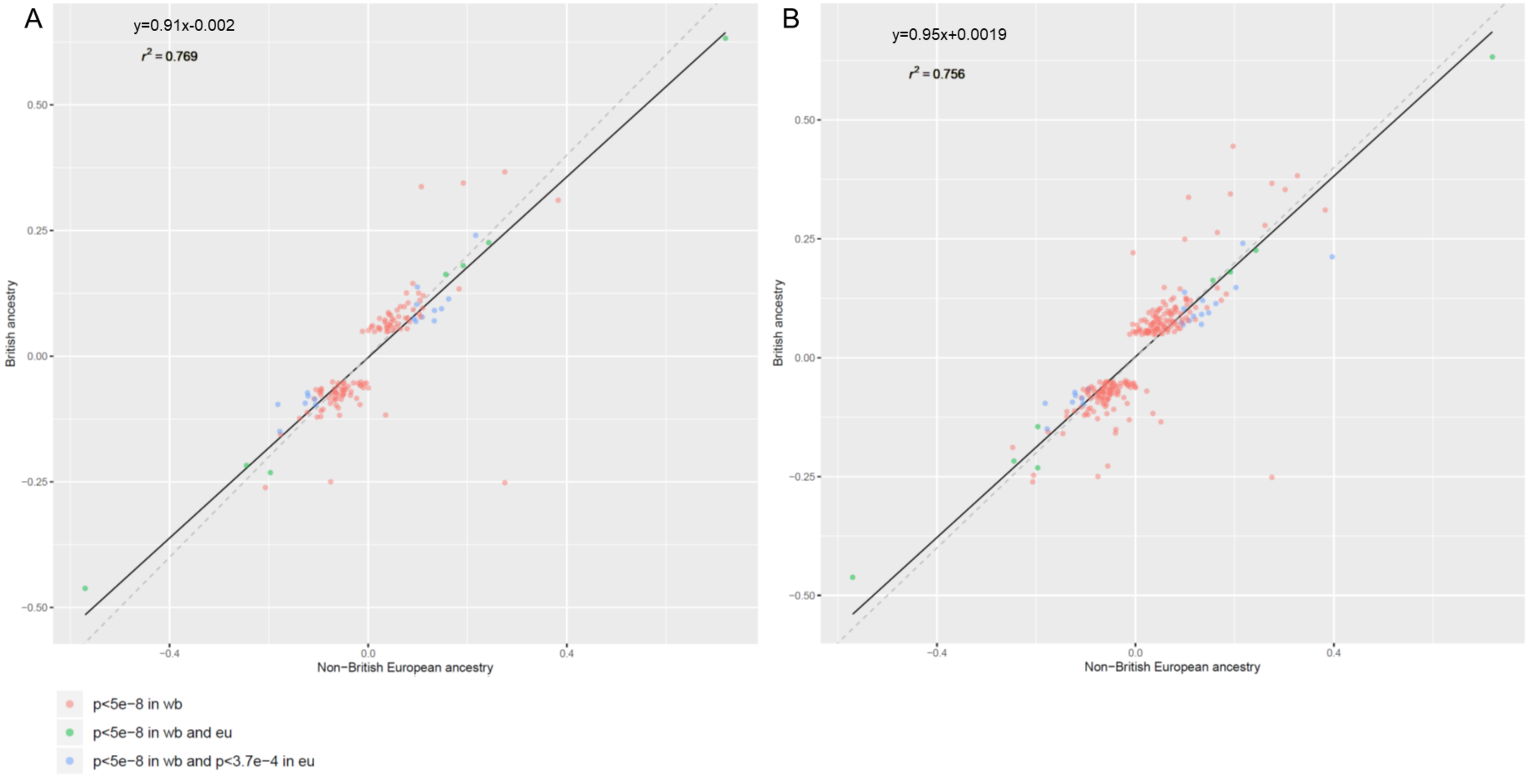

Supplementary Figure 2. CRF loci overlapping reported Fuchs endothelial cornea dystrophy risk loci. A. Regional association plots for three significant loci in a discovery GWAS using 1404 FECD cases and 2564 controls, reproduced from Afshari *et al* 2017[10]. B. Corresponding plots in the CRF GWAS analysis of white-British UKBB participants (N= 76029). Plots generated using LocusZoom display colour coded pair-wise linkage disequilibrium measure with reported FECD lead variants represented in boxes (with odds ratio reported for disease and effect on CRF in mmHg, both effects reported with 95% CI).

A.FECD association plots- from Afshari *et al* 2017[10]

B. Cornea resistance factor association

rs1200114 G/A OR 1.43 (1.29,1.57)

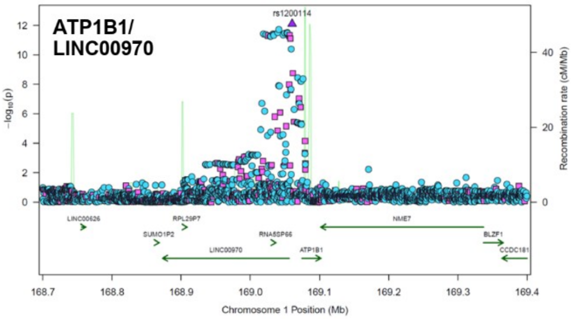

rs1200114 G/A -0.0973 (-0.1068,-0.0878)

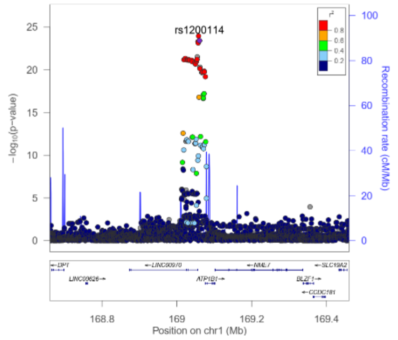

rs7104929 OR not reported

rs12223324 A/G OR=0.77(0.7,0.85)

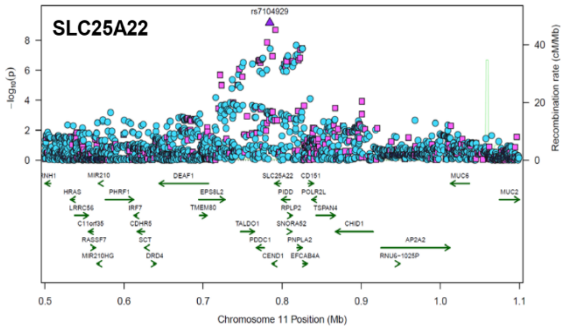

rs7104929 G/C -0.0921 (-0.1013,-0.0829)

rs12223324 A/G 0.0902 (0.0811,0.0993)

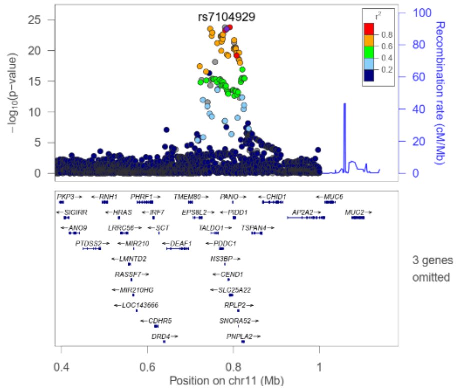

rs11659764 A/T OR 10.78 (9.06,12.83)

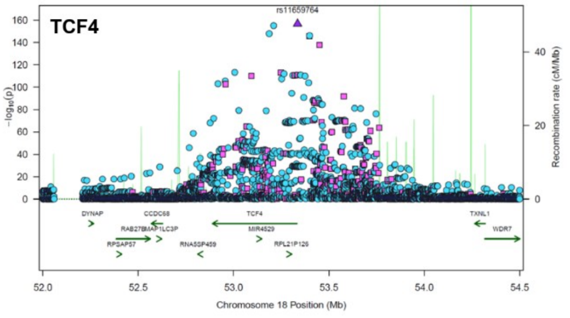

rs11659764 A/T -0.3373 (-0.3577,-0.3169)

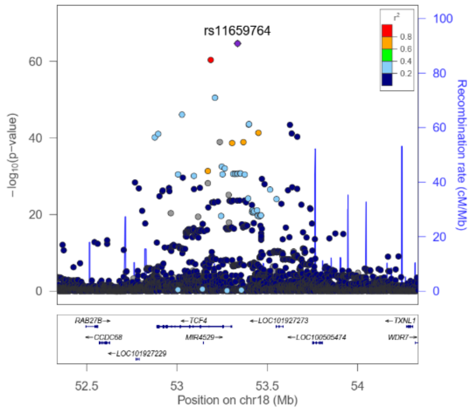

Supplementary Figure 3. Regulatory annotation enrichments using GARFIELD and default annotation resources (combined ENCODE, Roadmap epigenomics data).

Radial plot shows the enrichment (measured as an odds ratio and indicated by integer) in each cell type (bead on the outside of the circle sorted by tissue- font size of listed tissue proportional to its representation) for different GWAS significance thresholds[9](shown by inner colours and bottom legend). Small dots on the outer side of the plot show if the observed enrichment is significant (dot present) or not (if there is no dot) for thresholds 10^{-5},10^{-6},10^{-7},10^{-8} in direction from outside to inside.

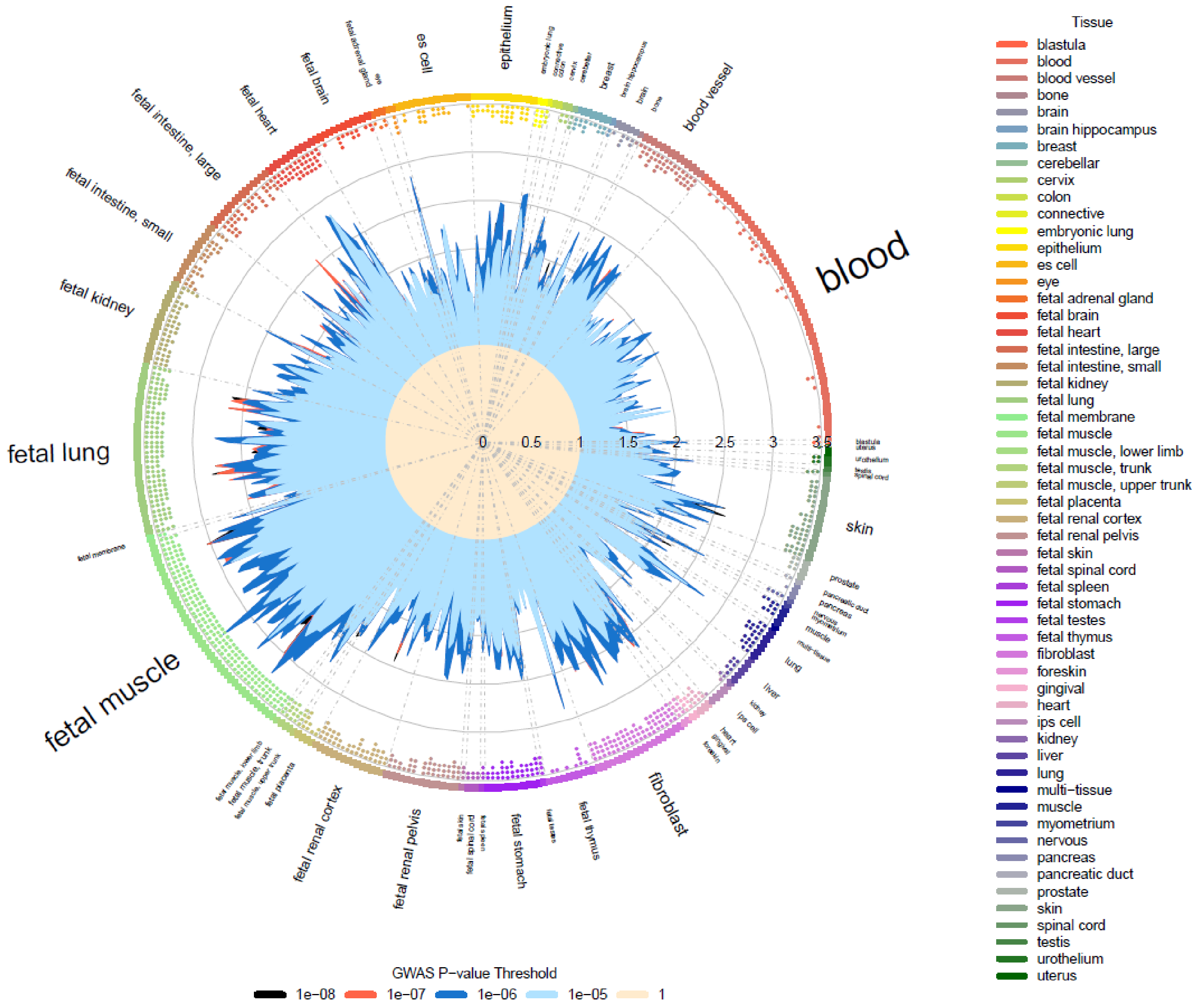

Supplementary Figure 4. ATAC-seq profiles in the immortalised corneal epithelial and stromal cell lines (respectively hTCEpi and hTK) around two non-coding variants with high causal probability used as tagging variants for open chromatin region enrichment analysis. A. around variant rs12913547, prioritised causal variant at locus 106- other variants from the same credible set and with similarly high causal probability indicated alongside. B. variant rs8127032, prioritised causal variant at locus 135, credible set 2-Screenshot from ucsc genome browser with derived annotations used in enrichment analysis in top tracks.

A.

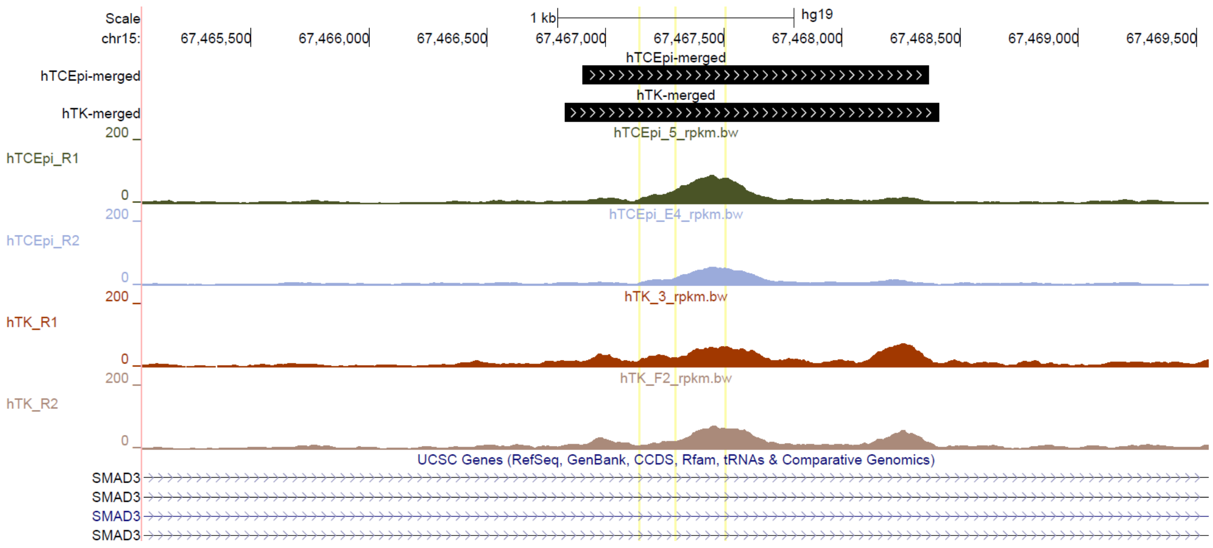

B.

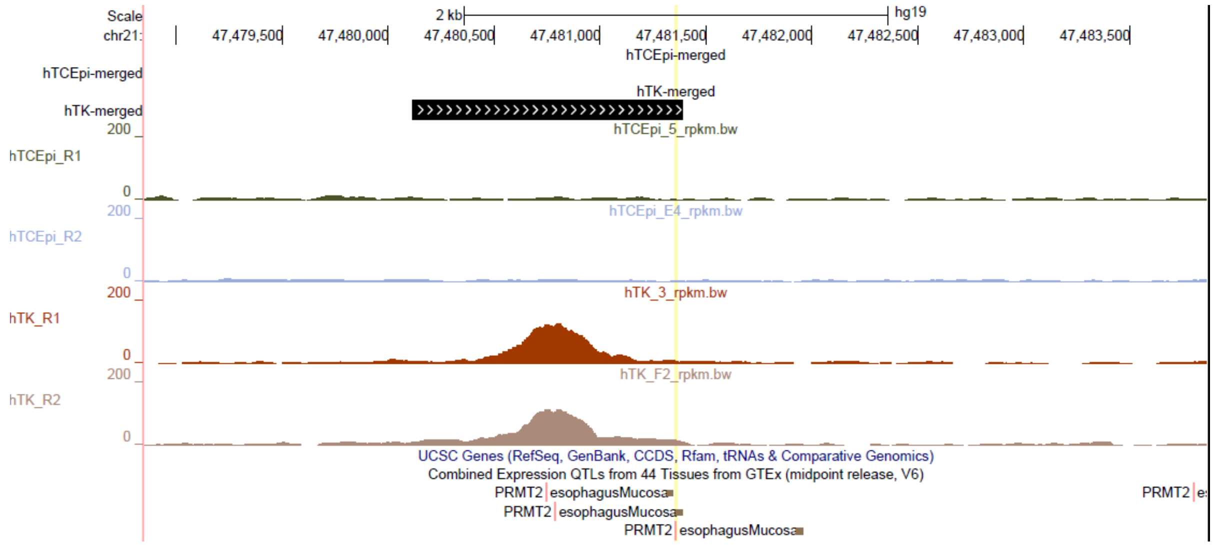

Supplementary Figure 5. Unsupervised clustering of individual ATAC-seq datasets evaluated for the CRF GWAS enrichment analysis. Publicly available datasets are listed Supplementary Data 11 ; RPE: retinal pigmented epithelium, CNCC: cranial neural crest cells, K562: myelogenous leukemia cell line; LCL: lymphoblastoid cell line; nDF: neonatal dermal fibroblast; DermF:adult dermal fibroblast; CEC: primary cornea epithelial cells. In-house generated datasets are from the immortalized cornea cell lines hTK and hTCEpi, performed in two replicates (rep1 and rep2).

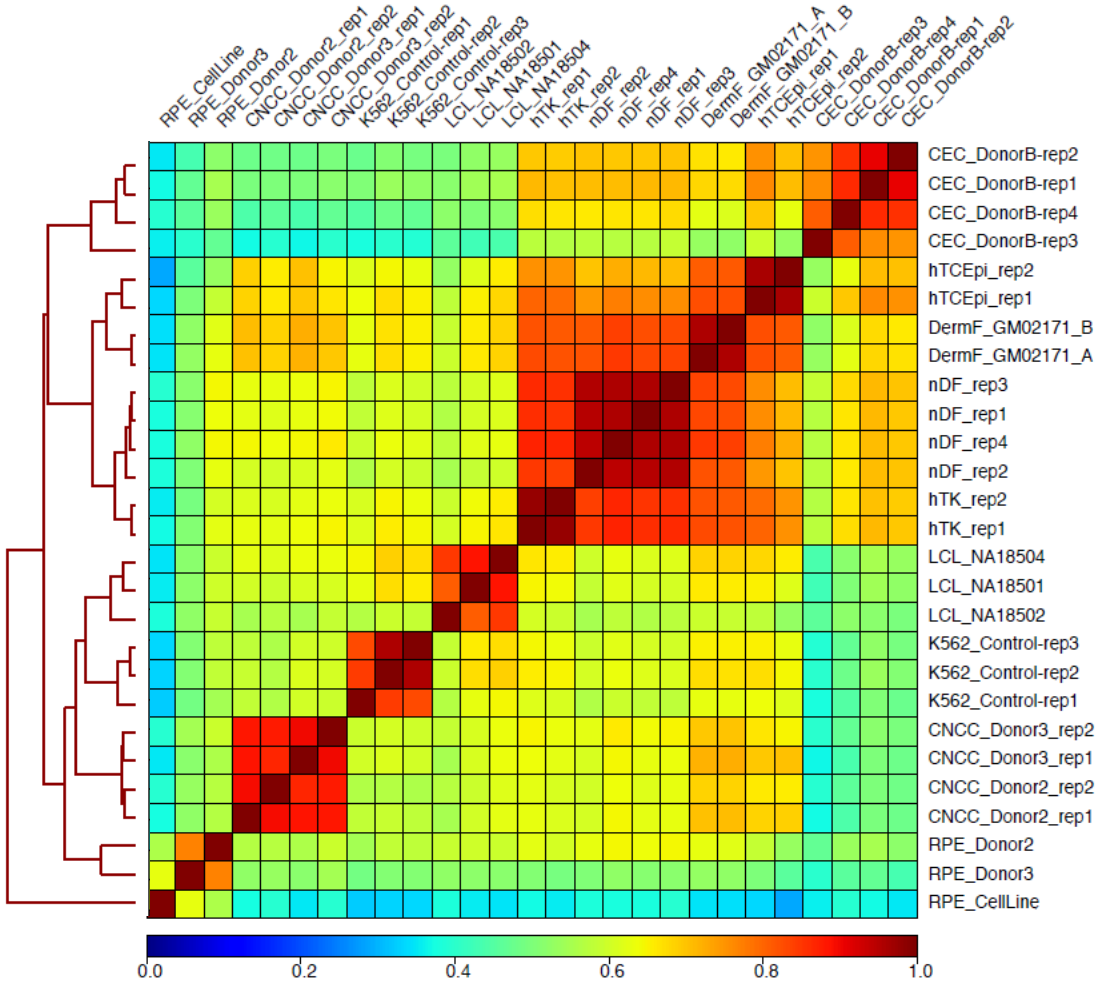

Supplementary Information References

1. Buenrostro, J.D., Wu, B., Chang, H.Y. & Greenleaf, W.J. ATAC-seq: A Method for Assaying Chromatin Accessibility Genome-Wide. *Curr Protoc Mol Biol* **109**, 21 29 1-9 (2015).
2. Wingett, S.W. & Andrews, S. FastQ Screen: A tool for multi-genome mapping and quality control. *F1000Res* **7**, 1338 (2018).
3. Amemiya, H.M., Kundaje, A. & Boyle, A.P. The ENCODE Blacklist: Identification of Problematic Regions of the Genome. *Sci Rep* **9**, 9354 (2019).
4. Li, H. *et al.* The Sequence Alignment/Map format and SAMtools. *Bioinformatics* **25**, 2078-9 (2009).
5. Quinlan, A.R. BEDTools: The Swiss-Army Tool for Genome Feature Analysis. *Curr Protoc Bioinformatics* **47**, 11 12 1-34 (2014).
6. Heinz, S. *et al.* Simple combinations of lineage-determining transcription factors prime cis-regulatory elements required for macrophage and B cell identities. *Mol Cell* **38**, 576-89 (2010).
7. Kent, W.J., Zweig, A.S., Barber, G., Hinrichs, A.S. & Karolchik, D. BigWig and BigBed: enabling browsing of large distributed datasets. *Bioinformatics* **26**, 2204-7 (2010).
8. Kuhn, R.M., Haussler, D. & Kent, W.J. The UCSC genome browser and associated tools. *Brief Bioinform* **14**, 144-61 (2013).
9. Iotchkova, V. *et al.* GARFIELD classifies disease-relevant genomic features through integration of functional annotations with association signals. *Nat Genet* **51**, 343-353 (2019).
10. Afshari, N.A. *et al.* Genome-wide association study identifies three novel loci in Fuchs endothelial corneal dystrophy. *Nat Commun* **8**, 14898 (2017).
11. Watanabe, K., Taskesen, E., van Bochoven, A. & Posthuma, D. Functional mapping and annotation of genetic associations with FUMA. *Nat Commun* **8**, 1826 (2017).
12. Iglesias, A.I. *et al.* Cross-ancestry genome-wide association analysis of corneal thickness strengthens link between complex and Mendelian eye diseases. *Nat Commun* **9**, 1864 (2018).
13. Ivarsdottir, E.V. *et al.* Sequence variation at ANAPC1 accounts for 24% of the variability in corneal endothelial cell density. *Nat Commun* **10**, 1284 (2019).
14. Choquet, H. *et al.* A multiethnic genome-wide analysis of 44,039 individuals identifies 41 new loci associated with central corneal thickness. *Commun Biol* **3**, 301 (2020).
15. Gao, X.R., Huang, H., Nannini, D.R., Fan, F. & Kim, H. Genome-wide association analyses identify new loci influencing intraocular pressure. *Hum Mol Genet* **27**, 2205-2213 (2018).
16. Khawaja, A.P. *et al.* Genome-wide analyses identify 68 new loci associated with intraocular pressure and improve risk prediction for primary open-angle glaucoma. *Nat Genet* **50**, 778-782 (2018).
17. Benner, C. *et al.* FINEMAP: efficient variable selection using summary data from genome-wide association studies. *Bioinformatics (Oxford, England)* **32**, 1493-501 (2016).
18. Coetzee, S.G., Coetzee, G.A. & Hazelett, D.J. motifbreakR: an R/Bioconductor package for predicting variant effects at transcription factor binding sites. *Bioinformatics* **31**, 3847-9 (2015).
