# Supplementary material for: Fine-mapping and cell-specific enrichment at corneal resistance factor loci prioritize candidate causal regulatory variants
Source: Commun Biol. 2020 Dec 11;3:762. doi: 10.1038/s42003-020-01497-w (PMC7732848; doi:10.1038/s42003-020-01497-w)
Supplement: Supplementary file 2 — Description of Additional Supplementary Items [file 42003_2020_1497_MOESM2_ESM.pdf]

Description of additional supplementary items

Supplementary Data 1. Cornea resistance factor GWAS loci following analysis of UK Biobank participants of white British ancestry. Previously reported CRF loci are denoted in bold. Boundaries are determined following the FUMA[11] linkage disequilibrium and distance-based algorithm using the default setting, with independent lead variants within the defined locus defined heuristically based on low linkage disequilibrium (r2 < 0.1). Positions are in build 37 of the human genome assembly. EA: effect allele; NEA: other allele; EAF: effect allele frequency; BETA: effect estimate (mmHg); SE: standard error of effect estimate; P: association P-value; NG: nearest gene; NGD: distance in bp to nearest gene; CCT GWAS loci: reported in Iglesias et al 2018[12] (IGGC, international Glaucoma Genetics consortium) and recent additions from Ivarsdottir et al 2019[13] (DECODE) and Choquet et al 2020[14] (GERA+IGGC); IOPg and IOPcc GWAS results for UKBB analyses reported in respectively Gao et al 2018[15] and Khawaja et al 2018[16]- interval overlapping or lead variant within genomic loci boundaries (start, end columns) or & indicates less than 100kb away.

Supplementary Data 2. Association summary statistics from CRF GWAS analysis using UK Biobank participants of European ancestry other than white British (EU) for lead SNPs in the white British ancestry (WB) CRF GWAS listed Supplementary Data1. Positions are in build 37 of the human genome assembly. EA: effect allele; NEA: other allele; EAF: effect allele frequency; BETA: effect estimate (mmHg); SE: standard error of effect estimate; P: association P-value.

Supplementary Data 3. Colocalisation probabilities for cornea resistance factor and central cornea thickness GWAS signals. pp0: posterior probability of no association with either trait; pp1: posterior probability of association with CRF, not with CCT; pp2: posterior probability of association with CCT, not with CRF; pp3: posterior probability of association with CRF and CCT, independent causal variants; pp4: posterior probability of association with CRF and CCT, shared causal variants; ppmax: highest posterior probability across hypotheses.

Supplementary Data 4. Candidate target genes associated with CRF. Candidate criteria are those from on-line tool FUMA[11] and based on proximity (posMapSNPs), eQTL evidence (eqtlMapSNPs) or chromatin interaction evidence (ciMap); gene annotations used ANNOVAR and GRCh37 human genome assembly.

Supplementary Data 5. Regions used for conditional and fine-mapping analyses. Regions defined in FUMA[11] using linkage disequilibrium measure r^2 threshold of 0.1 in both clumping steps (defining lead and independent SNPs)-start and end indicates locus coordinates under default FUMA setting (LD clumping threshold of 0.6 in second step). CRF GWAS results are those from the analysing the subset of unrelated white British UK Biobank participants; genomic locus naming is that of locus in the analysis of all participants-locus reaching genome-wide significance only in the unrelated individual subset are suffixed Ext.

Supplementary Data 6. Conditional and joint multiple SNPs analysis results. Signal contributing variant (SNP) per region are reported with their marginal effect (b; standard error, se; P-value, p) and effect in joint analysis (index J). * denotes sub-analysis-specific lead SNPs for which joint association p-value pJ drops just under genome-wide significance threshold.

Supplementary Data 7. Number of independent signals at CRF loci following different selection methods. Posterior probabilities for the number of independent causal signals are calculated following FINEMAP algorithm[17] -config_prob and credible sets for the most likely configuration displayed with top variant based on its inclusion probability (cred_set_X_prob) reported.

Supplementary Data 8. 95% credible sets determined by the Bayesian method implemented in the FINEMAP package. Credible set are named after the variant with the highest posterior inclusion probability (PIP). PIP and Bayes factor for causality in log 10 scale (log10(BF)) were obtained using FINEMAP[17] snp sss argument.

Supplementary Data 9. Functional annotation for subset of variants with strong statistical support of causing CRF association. PIP, posterior inclusion probability for 95% credible set of variants at independent causal signals calculated by FINEMAP[17]; log10(BF), evidence in favour of causality expressed as a Bayes factor.

Supplementary Data 10. Function of proteins implicated by likely causal missense coding variants. SNP, variant name; MAF, minor allele frequency in white British UK Biobank participants analysed; PIP, posterior probability of inclusion to list of candidate causal variants; CoJo, amongst selected variants following conditional and joint analysis using GCTA; CADD, Combined Annotation Dependent Depletion score; CCT: central cornea thickness, CH: cornea hysteresis; IOP: Intra-occular eye pressure, * SNP was reported as lead SNP.

Supplementary Data 11. Tally of transcription factor binding sites predicted to be disrupted at 30 CRF GWAS non-coding singly prioritized causal variant candidates. Transcription binding disruption potential was evaluated by the MotifBreakR package[18] with complete output displayed Supplementary Data 12. Highlighted cells correspond to transcription factor encoded by a candidate target genes listed Supplementary Data 4.

Supplementary Data 12. Complete information on transcription factors binding motifs disruption in silico predictions for each of the 30 prioritized CRF GWAS non-coding causal variant candidates. Output from MotifBreakR[18] is listed- pctRef or Alt represents the sequence surrounding the polymorphic site match to known transcrition factor motif as percentage of the best score the motif could achieve on an ideal sequence.

Supplementary Data 13. ATAC-seq datasets from the Gene Expression Omnibus (GEO) database considered for the enrichment analysis for corneal resistance factor GWAS variants. Shaded cells correspond to samples that were further merged prior to enrichment analysis. Summary of quality control that was performed on the older releases gives raw, processed and aligned to genome reference (hg19) read counts. ChrM represents mitochondrial contaminations, fragment quality qualifies the pattern of fragments lengths distribution presented Supplementary Note 4; label_enrichmentAnalysis represents the name for the dataset used in the enrichment analysis; Set for peak calling indicates whether this dataset was newly generated by merging datasets (in pool) or not.

Supplementary Data 14. Annotation information for ATAC-seq sets displaying significant CRF GWAS variants enrichments. Information is generated by the GARFIELD package[9], with positions in build 37. VAR_INFO indicates the GWAS variant selected based on P-value threshold (CRF association p-value, 0 if not located in an annotation or 1 if within annotation feature); INFO_TAGS_USED_TO_ANNOTATE represents variants in high LD ($r^2 > 0.8$) with selected variant; INFO_TAGS_PRUNED_OUT variants in lower LD with selected variant pruned out

Supplementary Data15. Annotation to an open-chromatin region in skin or keratocyte fibroblastic cells for eight of the prioritised causal variants underlying enrichment signals. Tagging variant is the GWAS variant selected (log10(BF) > 3) for functional annotation enrichment test. Map OCR denotes whether the selected (tagging) variant itself locates within the annotated OCR in respectively the skin (DermF) or corneal (hTK) fibroblastic cell line. Other tag with log10(BF) > 3 denotes variant in high LD (> 0.8) with tagging variant, and therefore with similarly high causal probability measured by Bayes factor (BF) and in the same credible set

(cred set). Cred set number refers to independent causal signal set as listed Supplementary Data 8. In bold: variants belonging to credible set listed column C. Shaded cells indicated OCR also in the hTK-specific set.

See the Supplementary Material for references.
